# Selective and sensitive visualization of endogenous nitric oxide in living cells and animals by a Si-rhodamine deoxylactam-based near-infrared fluorescent probe†
†Electronic supplementary information (ESI) available: Synthesis, experimental procedures, supplemental spectra and imaging data, and ^1^H-, ^13^C-NMR, and MS spectra. See DOI: 10.1039/c7sc02608k


**DOI:** 10.1039/c7sc02608k

**Published:** 2017-08-02

**Authors:** Yingying Huo, Junfeng Miao, Lingjun Han, Yaping Li, Zhe Li, Yawei Shi, Wei Guo

**Affiliations:** a School of Chemistry and Chemical Engineering , Shanxi University , Taiyuan 030006 , China . Email: guow@sxu.edu.cn; b Institute of Biotechnology , Shanxi University , Taiyuan 030006 , China; c Department of Chemistry , Taiyuan Normal University , Jinzhong 030619 , China

## Abstract

Nitric oxide (NO) is a fundamental signaling molecule that regulates virtually every critical cellular function, and it is also a potent mediator of cellular damage in a wide range of conditions mainly *via* its secondary metabolite peroxynitrite (ONOO^–^). In this work, we present an *o*-phenylenediamine (OPD)-locked Si-rhodamine deoxylactam, *i.e.***deOxy-DALSiR**, as a near-infrared fluorescent probe for the selective and sensitive detection of NO in living cells and bodies. Not only could the probe overcome the limitations suffered by widely used and commercialized OPD-type fluorescent NO probes, such as the possible interferences by dehydroascorbic acid/ascorbic acid/methylglyoxal (DHA/AA/MGO), pH-sensitive fluorescence output, and short excitation and emission wavelengths, but it can also avoid serious interference from cysteine (Cys) found in the rhodamine lactam-based fluorescent NO probes developed later. What’s more, the probe is fairly sensitive for NO, as evidenced by its rapid fluorescence response rate (within seconds), huge fluorescence off–on ratio (6300-fold), and ultra-low detection limit (0.12 nM). Its effectiveness and practicability have been demonstrated by the successful imaging of endogenous NO in RAW 264.7 macrophages, pancreatic β-cells, and endothelial EA.hy926 cells, as well as in inflamed and diabetic mouse models.

## Abstract 2

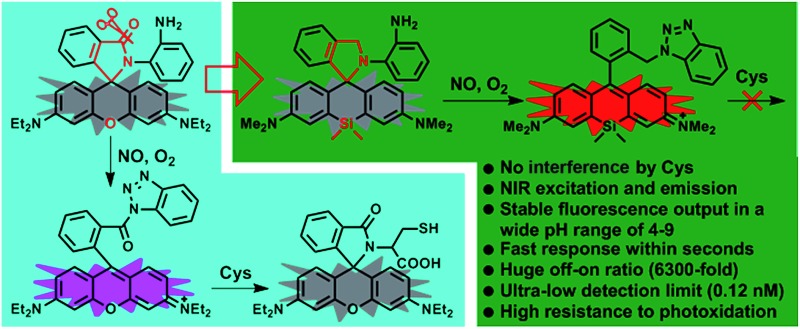
A Si-rhodamine deoxylactam-based near-infrared fluorescent probe has been successfully developed for the imaging of endogenous NO in living cells and mouse models.

## Introduction

Nitric oxide (NO), a ubiquitous messenger molecule in biological systems, is endogenously produced mainly by three NO synthases (eNOS, nNOS, and iNOS) from NADPH, O_2_ and l-arginine.^1^–^5^ NO produced *via* the eNOS and nNOS pathways is on the physiologic level, and plays various physiological roles in the human body, such as the inhibition of platelet aggregation and adhesion, blood vessel relaxation and angiogenesis, and neurotransmission. However, upon induction by the tumor necrosis factor TNF-α and lipopolysaccharide (LPS), a high level of intracellular NO could be produced *via* the iNOS pathway, which completely transforms the biological actions of NO by its fast reaction with a superoxide radical (O_2_^–^˙) to produce peroxynitrite (ONOO^–^), an extremely powerful oxidant.^6^–^8^ ONOO^–^, despite its positive functions in the immune response and redox regulation of cell signal,^6^ could severely damage a wide variety of molecular targets, including lipids, DNA, proteins and enzymes, ultimately leading to mitochondria dysfunction and cell death.^7^,^8^ Moreover, there is no biological defense system against ONOO^–^. As a result, ONOO^–^ has been implicated in a variety of disease states, including diabetes, Alzheimer’s disease, cancer, arthritis, autoimmune diseases, and other disorders.^4^ Thus, the development of methods and tools that can quickly, sensitively, and selectively track NO generation in biological systems is very important for unraveling its precise roles in health and disease.

Among various cellular biology tools, fluorescent probes, combined with fluorescence microscopy, have shown the unique advantages for mapping the spatial and temporal distributions of biological molecules in biological systems, due to their sensitivity, visualization, and noninvasiveness. Accordingly, in the past decade, a large number of fluorescent NO probes have been exploited, which typically utilize the specific reactions of NO with *o*-phenylenediamine (OPD)^9^–^17^ and metal–ligand complexes.^18^–^22^ Among them, OPD-based fluorescent NO probes were the earliest developed and are the most versatile fluorescent indicators for NO to date, and the corresponding sensing mechanism is based on the reaction of the OPD group with NO under aerobic conditions to form triazole derivatives, thereby turning on the fluorescence by inhibiting the photoinduced electron transfer (PeT) process. Even so, this type of probe suffers from some limitations, such as possible interference by dehydroascorbic acid/ascorbic acid/methylglyoxal (DHA/AA/MGO),^23^–^26^ pH-sensitive fluorescence output near neutral pH,^9^–^12^ and relatively long response time (commonly more than 5 min). As such, in recent years, some new strategies have been developed, such as diazo ring formation,^27^ NO-triggered ring-opening of the OPD-locked rhodamine lactam,^28^ reductive deamination of aromatic primary monoamines,^29^ the formation of Se–NO bonds,^30^ and the aromatization of Hantzsch ester.^31^ Although improved in many aspects, most of these probes still suffer from relatively long fluorescence response times, thus being unsatisfactory for real-time tracking of NO in biological systems (half-life of NO: 0.1–5 s). In addition, for tissue and living animal imaging, fluorescent probes with excitation and emission wavelengths in the near-infrared (NIR) region (650–900 nm) are especially favorable due to the high tissue penetration and low phototoxicity of NIR light, as well as the small background autofluorescence of biomacromolecules in the NIR region. However, as far as we know, only a few fluorescent NO probes could meet this requirement.^32^–^35^

In this work, we present an OPD-locked and Si-rhodamine deoxylactam-based fluorescent NO probe, *i.e.***deOxy-DALSiR**, which could not only overcome the above-mentioned limitations, as evidenced by its NIR excitation and emission wavelengths, negligible interference by DHA/AA/MGO, fast fluorescence response within seconds, and stable fluorescence output against pH changes, but also show some additional important sensing performances, including a baseline-level background fluorescence, huge fluorescence off–on ratio (6300-fold), and an ultra-low detection limit (0.12 nM). These features, combined with its good membrane permeability, low cytotoxicity, and high resistance to photoxidation, have made the probe an ideal indicator for the tracking of endogenous NO *in vitro* and *in vivo*.

## Results and discussion

### Design rationale and synthesis of **deOxy-DALSiR**

**Scheme 1:**
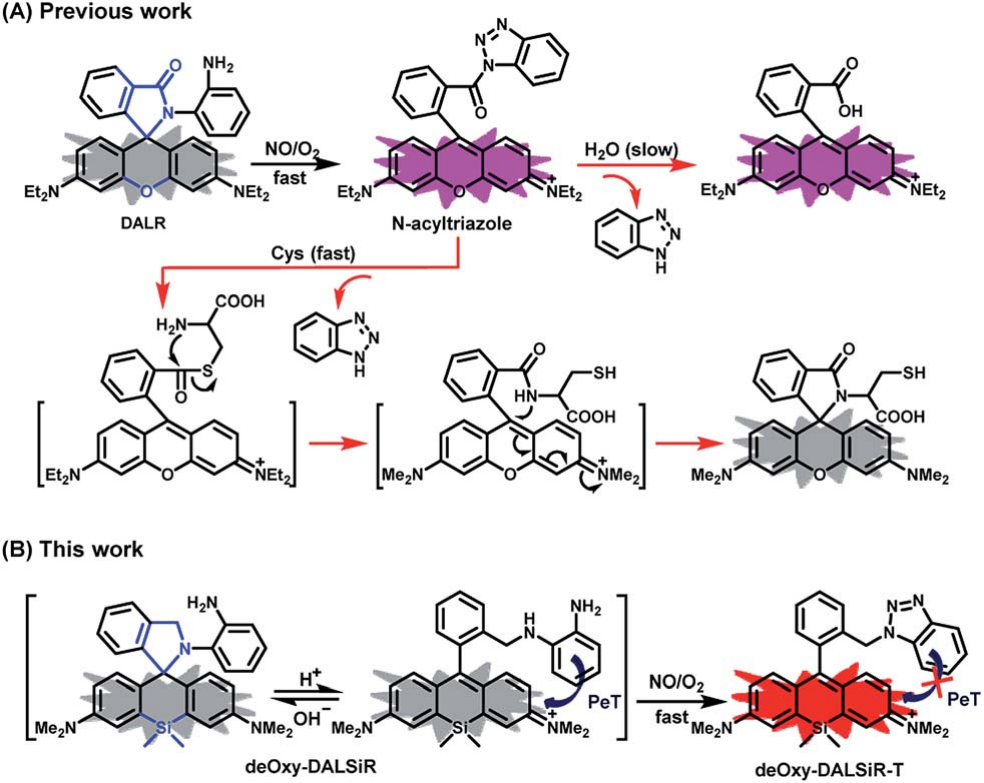
The strategies for the OPD-locked (Si)-rhodamine-based fluorescent NO probes.

The design of **deOxy-DALSiR** was inspired by OPD-locked rhodamine lactam (named **DALR** here), a fluorescent NO probe reported in 2008 (Scheme 1A).^28^ The easy synthesis, negligible background fluorescence, and big fluorescence off–on ratio have since made the probe an attractive platform for constructing various fluorescent NO probes.^34^,^36^–^40^ However, the *N*-acyltriazole intermediate, arising from the initial reaction of the probe with NO, suffers from slow hydrolysis, and as a result, the fluorescence response time of the probe for NO is relatively long (∼30 min). More seriously, although not detected in that report, the intermediate may suffer from a cysteine (Cys)-induced native-chemical-ligation (NCL) and cyclization cascade reaction^41^,^42^ leading to nonfluorescent rhodamine lactam (Scheme 1A), given that *N*-acyltriazoles are highly active for the *S*-acylation reaction^43^–^45^ and the resulting rhodamine lactam is highly stable in physiological pH.^46^ In fact, the possibility has been confirmed by our experimental results (see below). Thus, the sensitivity of the probe for intracellular NO would be discounted due to the relatively high physiological concentration of Cys in living cells (30–200 μM).^47^–^49^ Moreover, the visible excitation and emission wavelengths also make the probe unsuitable for *in vivo* imaging applications. We envisioned that the above limitations could be overcome by an OPD-locked Si-rhodamine deoxylactam derivative, *i.e.***deOxy-DALSiR** (Scheme 1B). According to our design principle, the reaction of **deOxy-DALSiR** with NO under aerobic conditions would bypass the *N*-acyltriazole intermediate to directly generate a stable *N*-alkyltriazole product **deOxy-DALSiR-T**, thereby avoiding interference from a water molecule or Cys. Not only that, but some additional important sensing advantages were also greatly expected for the probe: (1) it contains a potential NIR Si-rhodamine fluorophore,^50^ thus making it suitable for *in vivo* imaging applications; (2) it should have low background fluorescence at a physiological pH due to its spirocyclic structure, and, even if it exists in its ring-opened form in poorly acidic conditions, the low background fluorescence is also greatly expected due to PeT from the electron-rich OPD group to the excited Si-rhodamine fluorophore;^51^ (3) it should be able to resist interference by DHA/AA/MGO, because the locked or alkylated OPD group may lose its capability to condense with the three biological species to form fluorescent quinoxaline or the 1H-quinoxaline-2-one heterocycle;^23^–^26^ (4) it should respond rapidly to NO, because, unlike the OPD group of the widely used rhodamine or fluorescein-based fluorescent NO probes,^10^,^11^ the OPD group of the probe does not contain any electron-withdrawing substituent, thus making it highly reactive with NO;^32^ (5) it should show stable fluorescence for NO in a wide pH range, because its triazole product **deOxy-DALSiR-T** lacks any acidic NH proton, thereby precluding the triazolate-induced partial fluorescence quenching near neutral pH.^12^

**Scheme 2:**
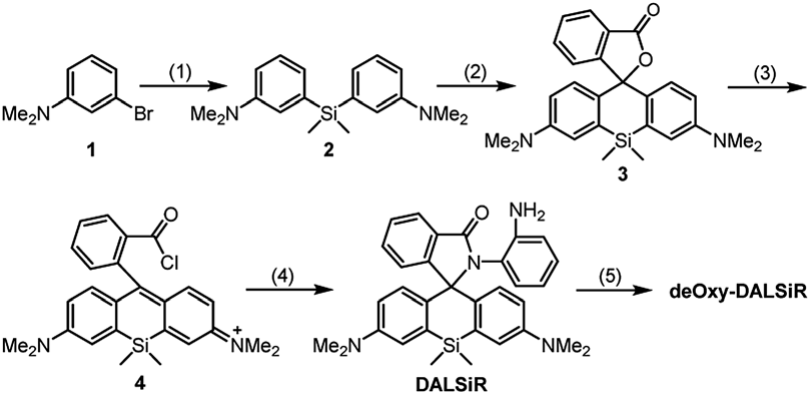
The synthesis of **deOxy-DALSiR**. (1) *n*-BuLi/Si(CH_3_)_2_Cl_2_; (2) 2-formylbenzoic acid/CuBr_2_; (3) POCl_3_; (4) OPD/NEt_3_; (5) BH_3_·THF.

With these considerations in mind, we synthesized **deOxy-DALSiR** starting from the commercially available 3-bromo-*N*,*N*-dimethylaniline **1**. As shown in Scheme 2, the reaction of **1** with *n*-BuLi in THF, followed by treatment with Si(CH_3_)_2_Cl_2_, gave diaryl silyl ether **2**; the treatment of **2** with 2-formylbenzoic acid in the presence of CuBr_2_ provided Si-rhodamine lactone **3**; the reaction of **3** with POCl_3_ in 1,2-dichloroethane gave Si-rhodamine chloride **4**; the reaction of **4** with OPD in the presence of NEt_3_ afforded the precursor **DALSiR**; the desired product **deOxy-DALSiR** was finally obtained by reducing **DALSiR** with BH_3_·THF solution. The detailed synthetic procedures are shown in the ESI.†

### The spectral response of **deOxy-DALSiR** to NO

**Fig. 1:**
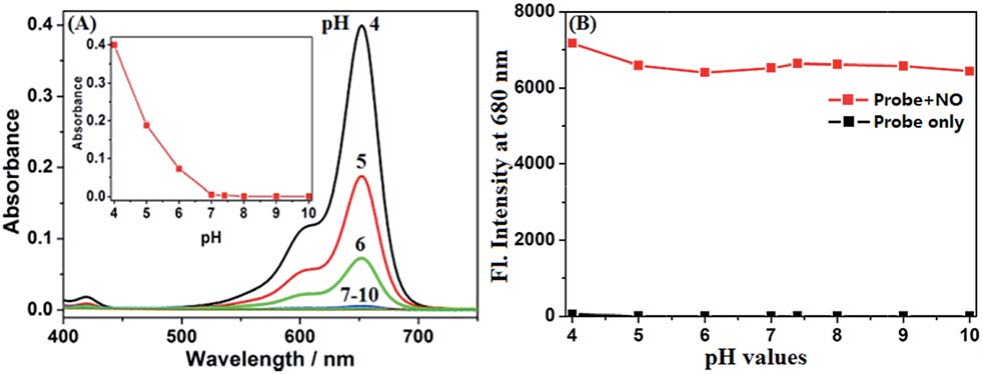
(A) The effect of pH on the absorption spectra of **deOxy-DALSiR** (4 μM). (B) The effect of pH on the fluorescence intensities of **deOxy-DALSiR** (4 μM) in the absence and presence of NO (30 μM). The conditions: B–R buffer (20 mM, pH = 4–10, containing 20% CH_3_CN); *λ*_ex_ = 645 nm; *λ*_em_ = 680 nm; slits: 5/10 nm.

It was reported that the ring-opening transformation of rhodamine deoxylactams is more sensitive to pH than that of rhodamine lactams.^52^,^53^ We envisioned that it should also be the case for **deOxy-DALSiR** due to the similar chemical structure. As shown in Fig. 1A, at pH = 4 **deOxy-DALSiR** showed a strong absorption peak at 650 nm, indicating that it exists mainly as the ring-opened form in poorly acidic conditions; as the pH values increased, the absorption intensities gradually decreased, and finally reached the baseline level when pH ≥ 7, indicating that its ring-closed form dominates in poorly basic conditions. Thus, the chemical structure of **deOxy-DALSiR** is pH-dependent. Notably, whether existing as the ring-closed form or as the ring-opened form, **deOxy-DALSiR** showed negligible fluorescence in the NIR region (Fig. 1B), consistent with previous speculation that **deOxy-DALSiR** is always nonfluorescent either due to its non-conjugated spirocyclic structure or the PeT quenching process (Scheme 1B). Importantly, in the pH region of 4–10, **deOxy-DALSiR** exhibited a dramatic fluorescence off–on response for NO in the NIR region (*λ*_max_ = 680 nm), strongly indicating that the probe has the potential to serve as an imaging tool for NO in complex biological environments.

**Fig. 2:**
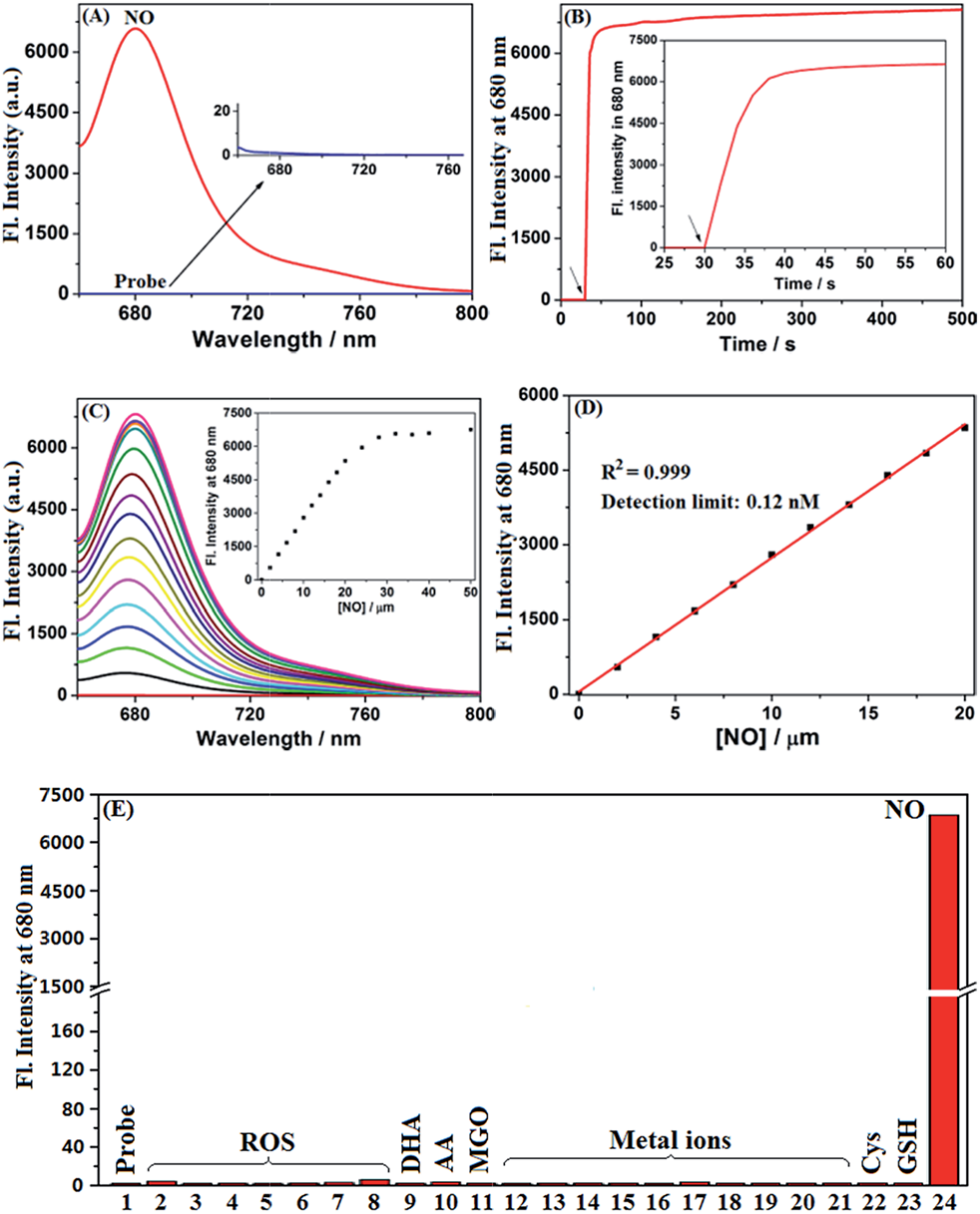
(A) The fluorescence spectra of **deOxy-DALSiR** (4 μM) before and after treatment with NO (30 μM). (B) The time course of the fluorescence intensities of **deOxy-DALSiR** (4 μM) at 680 nm after treatment with NO (30 μM). (C) The fluorescence spectra changes of **deOxy-DALSiR** (4 μM) treated with increasing concentrations of NO (0–50 μM). (D) The plot of the fluorescence intensities of **deOxy-DALSiR** (4 μM) at 680 nm as a function of the NO concentration (0–20 μM). (E) The fluorescence intensities of **deOxy-DALSiR** (4 μM) treated with various biologically relevant species for 5 min. (1) **deOxy-DALSiR** only; (2) HClO; (3) H_2_O_2_; (4) ^1^O_2_; (5) O_2_˙^–^; (6) ˙OH; (7) NO_2_^–^; (8) ONOO^–^; (9) DHA; (10) AA; (11) MGO; (12) K^+^; (13) Ca^2+^; (14) Na^+^; (15) Mg^2+^; (16) Al^3+^; (17) Zn^2+^; (18) Fe^2+^; (19) Fe^3+^; (20) Cu^+^; (21) Cu^2+^; (22) Cys; (23) GSH; (24) NO. The concentrations for (2–8), 100 μM; for (9–11), 1 mM; for (12–21), 100 μM; for (22), 200 μM; for (23) 1 mM; for (24), 30 μM. The conditions: PBS buffer (50 mM, pH 7.4, containing 20% CH_3_CN); *T* = 25 °C; *λ*_ex_ = 645 nm; *λ*_em_ = 680 nm; slits: 5/10 nm.

Encouraged by the above results, we thoroughly evaluated the fluorescence sensing performances of **deOxy-DALSiR** toward NO in the simulated physiological conditions (PBS, 50 mM, pH = 7.4, containing 20% CH_3_CN). As shown in Fig. 2A, the probe itself showed no observable emission when excited at 645 nm; upon treatment with excessive NO, a huge fluorescence off–on response up to 6300-fold was immediately observed at 680 nm. Remarkably, the fluorescence off–on response was considerably rapid and could be completed within seconds (Fig. 2B), indicative of the great potential of the probe for real-time tracing of NO generation in biosystems. Further, the fluorescence titration assay revealed a dose-dependent increase in the fluorescence intensities, which reached saturation when 30 μM NO was used (Fig. 2C). In this case, an excellent linear correlation between the fluorescence intensities and NO concentrations from 0 to 20 μM was achieved (Fig. 2D), and the detection limit was estimated to be as low as 0.12 nM based on 3*σ*/*k*. To the best of our knowledge, this is the highest detection sensitivity for NO achieved for fluorescent probes reported to date. Thus, it is very promising for the probe to detect trace amounts of intracellular NO. To establish the selectivity, we tested the fluorescence responses of the probe toward various biologically relevant species, including reactive oxygen species (ROS: ClO^–^, H_2_O_2_, O_2_˙^–^, ^1^O_2_, ˙OH, NO_2_^–^, and ONOO^–^), DHA/AA/MGO, metal ions (K^+^, Ca^2+^, Na^+^, Mg^2+^, Al^3+^, Zn^2+^, Fe^2+^, Fe^3+^, Cu^+^, and Cu^2+^), and Cys/GSH. As shown in Fig. 2E and S1 (ESI†), all of these competitive species, including DHA/AA/MGO, did not elicit any obvious fluorescence enhancement of the probe, suggesting that the probe possesses fairly high specificity for NO. Taken together, these results reveal that **deOxy-DALSiR** should outperform many of the previously reported fluorescent NO probes in terms of the greater fluorescence off–on ratio, faster fluorescence response rate, higher selectivity, and ultra-sensitivity.

To confirm the reaction mechanism, we carried out HPLC-MS assays on **deOxy-DALSiR** after treatment with NO. As shown in Fig. S2 (ESI†), the treatment of the probe with NO mainly produced a new product, which could be assigned to *N*-alkyltrizole **deOxy-DALSiR-T** based on the MS data (*m*/*z*: calcd 516.2578; found 516.2582). Further, we synthesized **deOxy-DALSiR-T** by treating **deOxy-DALSiR** with NaNO_2_ in a mixture of AcOH and methanol (ESI†), and found that its absorption and emission maxima and profiles are identical to those of **deOxy-DALSiR** treated with NO (Fig. S3, ESI†). Obviously, the results are in good agreement with our proposed reaction mechanism (Scheme 1B).

With **deOxy-DALSiR-T** in hand, we also tested the pH effects on its fluorescence intensities, given that the widely used OPD-based fluorescent NO probes commonly suffer from triazolate-induced partial fluorescence quenching *via* PeT near neutral pH.^9^–^12^ As shown in Fig. S4 (ESI†), due to the absence of any acidic NH proton in its *N*-alkyltriazole moiety, **deOxy-DALSiR-T** avoided the unfavorable deprotonated reaction, and thus displayed stable fluorescence in a wide pH range of 4–10.

### Comparison of the fluorescence sensing performances between **deOxy-DALSiR**, **DALSiR**, and **DALR**

**Fig. 3:**
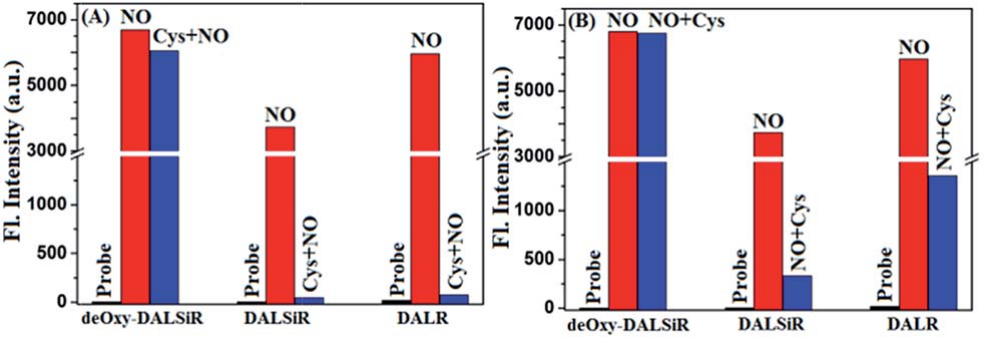
The effects of Cys (200 μM) on the fluorescence responses of **deOxy-DASiR**, **DASiR**, and **DAR** (all 4 μM) toward NO (80 μM for **deOxy-DASiR**; 400 μM for **DASiR** and **DAR**) in PBS buffer (50 mM, pH 7.4, containing 20% CH_3_CN) at 25 °C. The black bar: the fluorescence intensities of the probes. The red bar: the fluorescence intensities of the probes treated with NO after 1 min. The blue bar: (A) the fluorescence intensities of the probes treated with NO for 1 min in the presence of Cys; (B) the fluorescence intensities of the probes pre-treated with NO for 1 min and then treated with Cys for 10 min. For **deOxy-DASiR** and **DASiR**, *λ*_ex_ = 645 nm and *λ*_em_ = 680 nm; for **DAR**, *λ*_ex_ = 545 nm and *λ*_em_ = 582 nm; slits: 5/10 nm; voltage: 600 V.

It should be mentioned that **DALR** is a rhodamine lactam-based fluorescent NO probe that was reported in 2008 (Scheme 1),^28^ and **DALSiR** is a Si-rhodamine counterpart of **DALR** and also a synthetic precursor of **deOxy-DALSiR** (Scheme 2). To further illustrate the advantage of our design strategy, we compared the fluorescence sensing performances of **deOxy-DALSiR**, **DALSiR**, and **DALR** for NO in the presence of Cys, given that the *N*-acyltriazole intermediates resulting from the initial reactions of the latter two with NO may suffer from a Cys-induced NCL and cyclization cascade reaction to generate the nonfluorescent lactam products (Scheme 1A). As shown in Fig. 3A and S5 (ESI†), the addition of NO to the mixture of **deOxy-DALSiR** and Cys resulted in a nearly identical fluorescence off–on response to that in the absence of Cys, indicating that Cys hardly interfered with the reaction of **deOxy-DALSiR** with NO; by sharp contrast, almost no fluorescence response was observed when NO was added to the mixture of **DALSiR** (or **DALR**) and Cys, strongly indicating that the produced *N*-acyltriazole intermediate could be quickly consumed by Cys. Further, we performed a control assay by the initial treatment of the three compounds with NO, and then with Cys. As shown in Fig. 3B and S6 (ESI†), the fluorescence of **deOxy-DALSiR** treated with NO was not affected by the subsequent addition of Cys, whereas the fluorescence of **DALSiR** (or **DALR**) treated with NO dramatically decreased with the subsequent addition of Cys. The results further support the above speculation. Notably, the incomplete inhibition of the fluorescence in the cases of **DALSiR** or **DALR** (Fig. 3B) is presumably due to the lower amount of (Si-)rhodamine dye produced *via* the hydrolysis of the *N*-acyltriazole intermediate before Cys attack (Scheme 1A). In addition, the absorption spectra studies also support the above speculation. As shown in Fig. S7 (ESI†), **deOxy-DALSiR**, **DALSiR**, and **DALR** all showed negligible absorbance in the visible and NIR regions, due to their spirocyclic structures; upon the addition of NO, a strong absorption band was immediately observed for all of the three compounds, indicating that NO could trigger the ring-opening of the three compounds; after the subsequent treatment with Cys, the absorption intensity for the former remained almost unchanged, but for the latter two it decreased gradually within ten minutes, further supporting that the *N*-acyltriazole intermediate, resulting from the reaction of **DALSiR** (or **DALR**) with NO, could be captured by Cys. To confirm the reaction, we performed HPLC-MS assay on **DALR** pretreated with NO and then treated with Cys. As shown in Fig. S8 (ESI†), in addition to the lower amounts of unreacted **DALR**, a main new product was observed, which was proved to be the Cys-containing rhodamine lactam in terms of the MS data (calcd: 546.2427; found: 546.2423). Taken together, these results strongly suggest that the sensitivity of **deOxy-DALSiR** for intracellular NO should be superior to that of **DALSiR** or **DALR**, given the relatively high physiological concentration of Cys (30–200 μM) in mammal cells.^47^–^49^ In addition, we also tested the effects of some other biological reductants, such as GSH, Hcy, ascorbate (Asc), dithiothreitol (DTT), and NaHSO_3_, on the fluorescence response of **deOxy-DALSiR** toward NO. As shown in Fig. S9 (ESI†), the presence of these reductants virtually did not interfere with the fluorescence response of **deOxy-DALSiR** toward NO, consistent with the case of Cys.

### Imaging exogenous and endogenous NO in living cells using **deOxy-DALSiR**

**Fig. 4:**
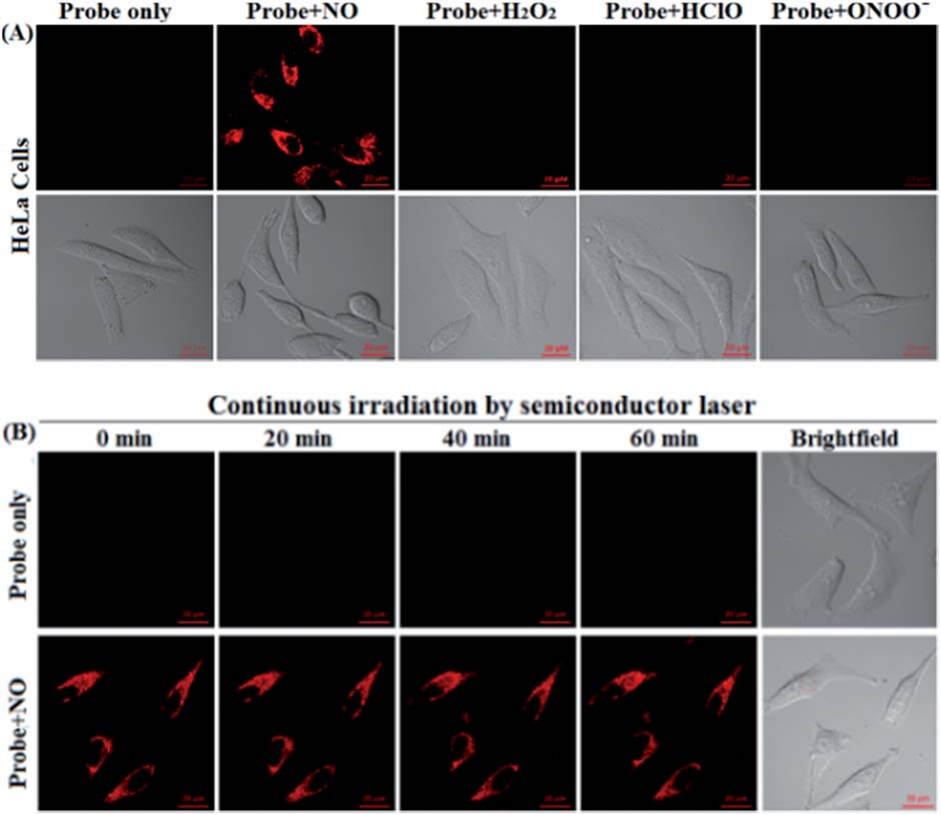
(A) The confocal images of the HeLa cells pretreated with **deOxy-DALSiR** (2 μM) for 20 min, and then treated with NOC-9 (20 μM), H_2_O_2_ (500 μM), ClO^–^ (500 μM), and SIN-1 (a ONOO^–^ donor: 500 μM) for 20 min in PBS. (B) The confocal images of the **deOxy-DALSiR** (2 μM)-loaded cells continuously irradiated by a semiconductor laser (633 nm) for 60 min in the absence and presence of NOC-9 (20 μM). The representative images were obtained at the indicated time points. The emission images were collected at 650–750 nm (*λ*_ex_: 633 nm). The scale bar: 20 μm.

Before the imaging assays, the cytotoxicity of **deOxy-DALSiR** and its alkyltrizole product **deOxy-DALSiR-T** was tested in HeLa cells by MTT assay. As shown in Fig. S10 (ESI†), after 24 h of cellular internalization of 2–50 μM **deOxy-DALSiR** (or **deOxy-DALSiR-T**), >90% of the cells remained viable, indicative of the excellent biocompatibility of both the probe and its alkyltrizole product. Even so, in order to reduce the interference to cell proliferation and physiology, a low concentration of **deOxy-DALSiR** (2 μM) was used in the subsequent bioimaging assays. Subsequently, the specificity of **deOxy-DALSiR** for NO in living cells was evaluated. As shown in Fig. 4A, when the HeLa cells were treated with **deOxy-DALSiR**, they showed almost no fluorescence; when the **deOxy-DALSiR**-loaded HeLa cells were treated with NOC-9 (a commercially available NO donor), strong intracellular fluorescence was observed in the red channel. Thus, the probe is cell membrane-permeable and could image exogenous NO in the cellular environment. Further, when the **deOxy-DALSiR**-loaded HeLa cells were treated with several representative ROS, such as H_2_O_2_, ClO^–^, and SIN-1 (a commercially available ONOO^–^ donor), respectively, almost no fluorescence was observed for each case, indicating that the probe still possesses high specificity for NO in cellular environments. Notably, the continuous irradiation of the **deOxy-DALSiR**-loaded HeLa cells in the absence and presence of NOC-9 in the imaging conditions for 60 min neither elicited any obvious fluorescence enhancement for the former, nor resulted in any obvious fluorescence decrease for the latter (Fig. 4B, details in Fig. S11, and ESI Videos S1 and S2†). Thus, **deOxy-DALSiR** and its trizole product could tolerate photoxidation and photobleaching, respectively, thus being suitable for time-lapse and long-term bioimaging applications.

**Fig. 5:**
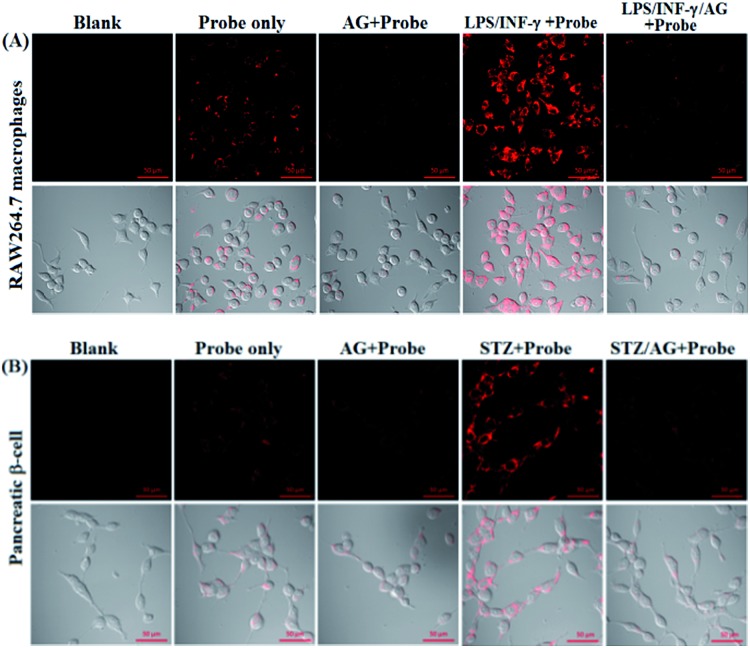
The confocal images of NO in the RAW 264.7 macrophages (A) and pancreatic β-cells (B) under different conditions. For imaging of intracellular basal NO, the cells were treated with **deOxy-DALSiR** (2 μM, 20 min) only; for imaging of stimulator-induced NO, the cells were pretreated with stimulators LPS (20 μg mL^–1^, 6 h)/INF-γ (150 units per mL, 6 h) (A) or STZ (0.5 mM, 12 h) (B), and then treated with **deOxy-DALSiR** (2 μM, 20 min); for the inhibition assays, the cells were pretreated with LPS (20 μg mL^–1^, 6 h)/INF-γ (150 units per mL, 6 h) or STZ (0.5 mM, 12 h) in the presence of AG (0.5 mM) and then treated with **deOxy-DALSiR** (2 μM, 20 min). The emission images were collected at 650–750 nm (*λ*_ex_: 633 nm). The scale bar: 50 μm.

Encouraged by the above results, we explored the potential applications of **deOxy-DALSiR** for the imaging of endogenous NO in living cells. The assays were first performed in RAW264.7 macrophages because these cells are known to express high-level iNOS upon stimulation by LPS/INF-γ.^54^,^55^ As shown in Fig. 5A, the cells themselves were nonfluorescent; when the cells were incubated with **deOxy-DALSiR**, a weak but clear intracellular fluorescence was observed in the red channel; when the cells were pretreated with the NO synthase inhibitor aminoguanidine (AG)^56^ and then treated with **deOxy-DALSiR**, the intracellular fluorescence greatly decreased; when the cells were stimulated with LPS/INF-γ and then treated with **deOxy-DALSiR**, a dramatic fluorescence enhancement in the red channel was observed; when the cells were stimulated with LPS/INF-γ in the presence of AG and then treated with **deOxy-DALSiR**, only weak intracellular fluorescence was observed. Thus, **deOxy-DALSiR** could image not only the basal NO but also the stimulation-induced NO in RAW264.7 cells, consistent with its high sensitivity found in chemical conditions. Further, the probe was utilized to monitor endogenous NO in pancreatic β-cells (INS-1), given that the excessive and sustained generation of NO derived from iNOS plays an important role in pancreatic β-cell death and the pathophysiological progression of diabetes.^57^,^58^ In the assays, streptozotocin (STZ), a widely used chemical to trigger pancreatic β-cell damage and induce experimental diabetes by the production of ROS and NO,^57^ was used as the inducer. As shown in Fig. 5B, similar to the case in the RAW264.7 macrophages, the probe could image both the basal and STZ-induced NO in pancreatic β-cells; moreover, the STZ-induced NO generation in pancreatic β-cells is dose-dependent and temporally regulated (Fig. S12, ESI†). Thus, **deOxy-DALSiR** holds great potential for studying diabetes pathogenesis.

**Fig. 6:**
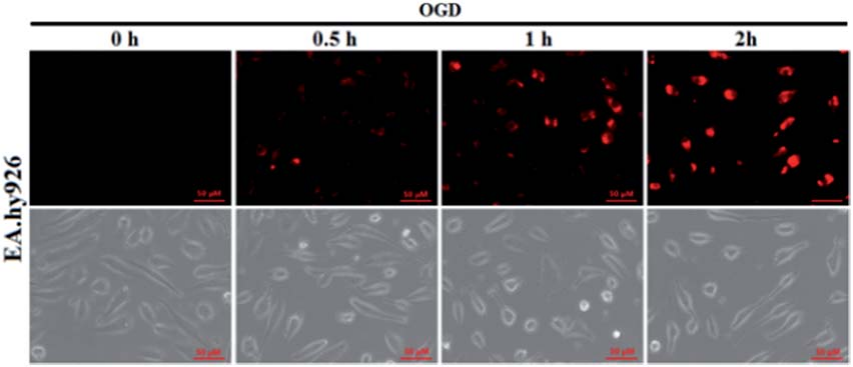
The time-dependent fluorescence accumulation of **deOxy-DALSiR** (2 μM) in EA.hy926 endothelial cells over 0.5 to 2 h following OGD exposure. The images were obtained using DeltaVision Microscopy Imaging Systems, and the excitation and emission bandpasses of the standard Cy5 filter set were used. The scale bar: 50 μm.

It is known that ischemia-reperfusion leads to increased iNOS-mediated NO production and the subsequent formation of ONOO^–^*via* the diffusion-controlled reaction of NO and O_2_˙^–^.^59^ ONOO^–^ is a powerful oxidant and its overproduction during ischemia could cause severe damage to endothelial cells.^60^–^62^ Thus, we also performed the assay of visualizing NO production using **deOxy-DALSiR** in endothelial EA.hy926 cells after oxygen-glucose deprivation (OGD), a widely used *in vitro* ischemic model.^63^–^65^ As shown in Fig. 6, a time-dependent fluorescence enhancement was observed in the endothelial cells over 0.5 to 2 h following OGD exposure, revealing that the probe is competent enough to monitor NO fluxes during ischemia.

**Fig. 7:**
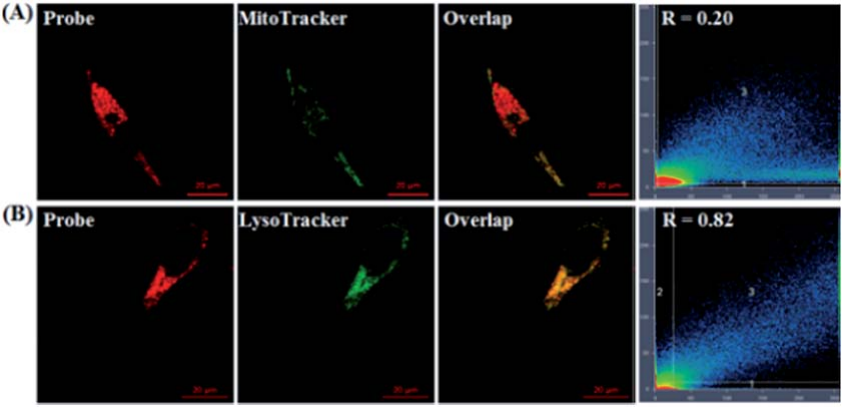
The fluorescence images of the HeLa cells co-stained with **deOxy-DALSiR** (2 μM) and MitoTracker green FM (0.2 μM) (A) or LysoTracker Green DND-26 (0.07 μM) (B), and then treated with NOC-9 (20 μM). For **deOxy-DALSiR**, the emission images were collected at 650–750 nm (*λ*_ex_: 633 nm); for MitoTracker or LysoTtracker, the emission was at 500–600 nm (*λ*_ex_: 488 nm).

To probe the subcellular localization of **deOxy-DALSiR**, we performed costaining assays in HeLa cells. In the assays, NOC-9 was used to light up the probe in the cells, and Pearson’s correlation coefficient (*R*) was used to analyze the scope of distribution between the two fluorescent channels from the probe and commercial trackers. As shown in Fig. 7A, when the cells were costained with **deOxy-DALSiR** and a commercial MitoTracker followed by NOC-9 treatment, a poor overlapping image and low Pearson’s correlation coefficient (*R* = 0.20) were observed. By contrast, when the cells were costained with **deOxy-DALSiR** and commercial LysoTracker followed by NOC-9 treatment, a good overlapping image and high Pearson’s correlation coefficient (*R* = 0.82) were found (Fig. 7B), indicating that the probe was mainly located in the lysosomes. A feasible explanation is that **deOxy-DASiR** is easy to protonate in poorly acidic conditions (Fig. 1A), and thus could be trapped by the poorly acidic lysosomes (pH range: 4.5–5.5), consistent with the proton-driving lysosome localization of the alkylmorpholine-containing lysosomal probes.^66^ The result is interesting because NO has been reported to play important roles in lysosome-related disorders and diseases, including lysosomal storage disorders,^67^ Gaucher’s disease,^68^ and Danon disease,^69^ and there are few fluorescent NO probes that can target lysosomes so far.^17^,^35^

### *In vivo* imaging of NO generation in mouse models using **deOxy-DALSiR**

**Fig. 8:**
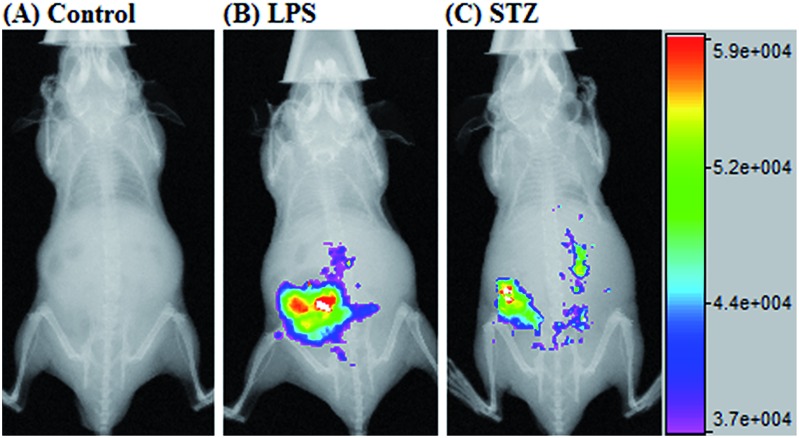
Overlay of the fluorescence images and X-ray images of mice. (A) The mouse was i.p. injected with **deOxy-DALSiR** (100 μL, 2 μM) for 30 min. (B) The mouse was first i.p. injected with LPS (1 mg mL^–1^, 100 μL) for 24 h, and then i.p. injected with **deOxy-DALSiR** (2 μM, 100 μL) for 30 min. (C) The mouse was first i.p. injected with STZ (40 mg kg^–1^) for 24 h, and then i.p. injected with **deOxy-DALSiR** (100 μL, 2 μM) for 30 min.

Given that **deOxy-DALSiR** could operate in the NIR region, we further investigated its potential to image endogenous NO in living nude mice. The images were obtained using a Bruker In-Vivo FX Pro small animal optical imaging system with an excitation filter of 620 nm and an emission filter of 670 nm. As can be seen in Fig. 8A, when the mouse was intraperitoneally (i.p.) injected with **deOxy-DALSiR** for 30 min, almost no fluorescence signal was observed; when the mouse was i.p. injected with LPS for 12 h to induce inflammation, followed by i.p. injection of **deOxy-DALSiR**, an obvious fluorescence signal was observed in the abdominal region (Fig. 8B), indicating that **deOxy-DALSiR** could image endogenous NO in the inflamed mouse model. Further, we tested the *in vivo* sensing performance of **deOxy-DALSiR** for NO in the STZ-treated mouse model. As shown in Fig. 8C, when the mouse was i.p. injected with STZ for 12 h, followed by i.p. injection of **deOxy-DALSiR**, a clear fluorescence signal was also observed in the abdominal region, consistent with iNOS being highly expressed in the initial stage of diabetes.^57^,^58^ Thus, **deOxy-DALSiR** holds great potential for studying the pathological roles of NO in living animals.

## Conclusions

In summary, we reported an OPD-locked, Si-rhodamine deoxylactam-based near-infrared fluorescent NO probe **deOxy-DALSiR**. The probe not only overcame the limitations suffered by most of the previously reported OPD-based fluorescent NO probes, such as possible interference by DHA/AA/MGO, slow response rate, pH-sensitive fluorescence output, and short excitation and emission wavelengths, but also avoided the severe interference from Cys suffered by the OPD-locked and rhodamine lactam-based fluorescent NO probes developed later. More importantly, the probe could detect NO with a rapid response rate, huge fluorescence off–on ratio, and ultra-low detection limit. These excellent sensing performances, coupled with the good cell permeability and low cytotoxicity, have enabled the probe to image endogenous NO not only in RAW 264.7 macrophages, pancreatic β-cells, and endothelial EA.hy926 cells, but also in living mouse models. The probe is greatly expected to be a useful imaging tool for studying NO-related physiological and pathological functions.

## Supplementary Material

Supplementary information

Supplementary movie

Supplementary movie

## References

[cit1] Nitric Oxide, Handbook of Experimental Pharmacology, ed. B. Mayer, Springer, Berlin, 2000, vol. 143.

[cit2] Nitric Oxide Biology and Pathobiology, ed. L. J. Ignarro, Academic Press, San Diego, CA, 2000.

[cit3] Wink D. A., Mitchell J. B. (1998). Free Radical Biol. Med..

[cit4] Pacher P., Beckman J. S., Liaudet L. (2007). Physiol. Rev..

[cit5] de Mel A., Murad F., Seifalian A. M. (2011). Chem. Rev..

[cit6] Liaudet L., Vassalli G., Pacher P. (2009). Front. Biosci..

[cit7] Radi R. (2013). J. Biol. Chem..

[cit8] Ferrer-Sueta G., Radi R. (2009). ACS Chem. Biol..

[cit9] Kojima H., Nakatsubo N., Kikuchi K., Kawahara S., Kirino Y., Nagoshi H., Hirata Y., Nagano T. (1998). Anal. Chem..

[cit10] Kojima H., Urano Y., Kikuchi K., Higuchi T., Hirata Y., Nagano T. (1999). Angew. Chem., Int. Ed..

[cit11] Kojima H., Hirotani M., Nakatsubo N., Kikuchi K., Urano Y., Higuchi T., Hirata Y., Nagano T. (2001). Anal. Chem..

[cit12] Gabe Y., Urano Y., Kikuchi K., Kojima H., Nagano T. (2005). J. Am. Chem. Soc..

[cit13] Sasaki E., Kojima H., Nishimatsu H., Urano Y., Kikuchi K., Hirata Y., Nagano T. (2005). J. Am. Chem. Soc..

[cit14] Zhang X., Wang H., Li J.-S., Zhang H.-S. (2003). Anal. Chim. Acta.

[cit15] Huang K.-L., Wang H., Ma M., Zhang X., Zhang H.-S. (2007). Nitric Oxide.

[cit16] Zhang H.-X., Chen J.-B., Guo X.-F., Wang H., Zhang H.-S. (2014). Anal. Chem..

[cit17] Yu H., Xiao Y., Jin L. (2012). J. Am. Chem. Soc..

[cit18] Lim M. H., Lippard S. J. (2005). J. Am. Chem. Soc..

[cit19] Lim M. H., Wong B. A., Pitcock Jr W. H., Mokshagundam D., Baik M.-H., Lippard S. J. (2006). J. Am. Chem. Soc..

[cit20] Lim M. H., Xu D., Lippard S. J. (2006). Nat. Chem. Biol..

[cit21] Pluth M. D., McQuade L. E., Lippard S. J. (2010). Org. Lett..

[cit22] McQuade L. E., Ma J., Lowe G., Ghatpande A., Gelperin A., Lippard S. J. (2010). Proc. Natl. Acad. Sci. U. S. A..

[cit23] Zhang X., Kim W.-S., Hatcher N., Potgieter K., Moroz L. L., Gillette R., Sweedler J. V. (2002). J. Biol. Chem..

[cit24] Ye X., Rubakhin S. S., Sweedler J. V. (2008). J. Neurosci. Methods.

[cit25] Wang T., Douglass Jr E. F., Fitzgerald K. J., Spiegel D. A. (2013). J. Am. Chem. Soc..

[cit26] Wang S.-T., Lin Y., Spicer C. D., Stevens M. M. (2015). Chem. Commun..

[cit27] Yang Y., Seidlits S. K., Adams M. M., Lynch V. M., Schmidt C. E., Anslyn E. V., Shear J. B. (2010). J. Am. Chem. Soc..

[cit28] Zheng H., Shang G.-Q., Yang S.-Y., Gao X., Xu J.-G. (2008). Org. Lett..

[cit29] Shiue T.-W., Chen Y.-H., Wu C.-M., Singh G., Chen H.-Y., Hung C.-H., Liaw W.-F., Wang Y.-M. (2012). Inorg. Chem..

[cit30] Sun C., Shi W., Song Y., Chen W., Ma H. (2011). Chem. Commun..

[cit31] Ma S., Fang D.-C., Ning B., Li M., He L., Gong B. (2014). Chem. Commun..

[cit32] Sasaki E., Kojima H., Nishimatsu H., Urano Y., Kikuchi K., Hirata Y., Nagano T. (2005). J. Am. Chem. Soc..

[cit33] Terai T., Urano Y., Izumi S., Kojima H., Nagano T. (2012). Chem. Commun..

[cit34] Gong Y.-J., Zhang X.-B., Mao G.-J., Su L., Meng H.-M., Tan W., Feng S., Zhang G. (2016). Chem. Sci..

[cit35] Wang B., Yu S., Chai X., Li T., Wu Q., Wang T. (2016). Chem.–Eur. J..

[cit36] Yu H., Jin L., Dai Y., Li H., Xiao Y. (2013). New J. Chem..

[cit37] Yu H., Zhang X., Xiao Y., Zou W., Wang L., Jin L. (2013). Anal. Chem..

[cit38] Yuan L., Lin W., Xie Y., Chen B., Zhu S. (2012). J. Am. Chem. Soc..

[cit39] Li Y., Wu W., Yang J., Yuan L., Liu C., Zheng J., Yang R. (2016). Chem. Sci..

[cit40] Wang C., Song X., Han Z., Li X., Xu Y., Xiao Y. (2016). ACS Chem. Biol..

[cit41] Yang X.-F., Huang Q., Zhong Y., Li Z., Li H., Lowry M., Escobedo J. O., Strongin R. M. (2014). Chem. Sci..

[cit42] Liu J., Sun Y.-Q., Zhang H., Huo Y., Shi Y., Shi H., Guo W. (2014). RSC Adv..

[cit43] Katritzky A. R., Ibrahim T. S., Tala S. R., Abo-Dya N. E., Abdel-Samii Z. K., RI-Feky S. A. (2011). Synthesis.

[cit44] Katritzky A. R., Angrish P., Todadze E. (2009). Synlett.

[cit45] Sun Y.-Q., Liu J., Zhang H., Huo Y., Lv X., Shi Y., Guo W. (2014). J. Am. Chem. Soc..

[cit46] Chen X., Pradhan T., Wang F., Kim J. S., Yoon J. (2012). Chem. Rev..

[cit47] Tateishi N., Higashi T., Naruse A., Nakashima K., Shiozaki H., Sakamoto Y. (1977). J. Nutr..

[cit48] Chung T. K., Funk M. A., Baker D. H. (1990). J. Nutr..

[cit49] Park S., Imlay J. A. (2003). J. Bacteriol..

[cit50] Sun Y.-Q., Liu J., Lv X., Liu Y., Zhao Y., Guo W. (2012). Angew. Chem., Int. Ed..

[cit51] Koide Y., Urano Y., Hanaoka K., Terai T., Nagano T. (2011). ACS Chem. Biol..

[cit52] Best Q. A., Xu R., McCarroll M. E., Wang L., Dyer D. J. (2010). Org. Lett..

[cit53] Li Z., Song Y., Yang Y., Yang L., Huang X., Han J., Han S. (2012). Chem. Sci..

[cit54] Lorsbach R. B., Murphy W. J., Lowenstein C. J., Snyder S. H., Russell S. W. (1993). J. Biol. Chem..

[cit55] Iovine N. M., Pursnani S., Voldman A., Wasserman G., Blaser M. J., Weinrauch Y. (2008). Infect. Immun..

[cit56] Muijsers R. B. R., van den Worm E., Folkerts G., Beukelman C. J., Koster A. S., Postma D. S., Nijkamp F. P. (2000). Br. J. Pharmacol..

[cit57] Zhang Y., Mei H., Shan W., Shi L., Chang X., Zhu Y., Chen F., Han X. (2016). J. Cell. Mol. Med..

[cit58] Chi Q., Wang T., Huang K. (2005). Biochem. Biophys. Res. Commun..

[cit59] Prathapasinghe G. A., Siow Y. L., Xu Z., O K. (2008). Am. J. Physiol. Renal Physiol..

[cit60] Dugo L., Serraino I., Fulia F., De Sarro A., Caputi A. P., Cuzzocrea S. (2001). J. Pineal Res..

[cit61] Teixeira A., Morfim M. P., de Cordova C. A., Charão C. C., de Lima V. R., Creczynski T. B. (2003). J. Pineal Res..

[cit62] Yin J., Liu Y. H., Xu Y. F., Zhang Y. J., Chen J. G., Shu B. H., Wang J. Z. (2006). J. Pineal Res..

[cit63] Golderg M. P., Choi D. W. (1993). J. Neurosci..

[cit64] Shi H., Liu S., Miyake M., Liu K. J. (2006). Chem. Res. Toxicol..

[cit65] Yang W., Will Thompson J., Wang Z., Wang L., Sheng H., Foster M. W. (2012). J. Proteome Res..

[cit66] Xu W., Zeng Z., Jiang J.-H., Chang Y.-T., Yuan L. (2016). Angew. Chem., Int. Ed..

[cit67] Ballabio A., Gieselmann V. (2009). Biochim. Biophys. Acta, Mol. Cell Res..

[cit68] Pavlova E. V., Deegan P. B., Tindall J., McFarlane I., Mehta A., Hughes D., Wraith J. E., Cox T. M. (2011). Blood Cells, Mol., Dis..

[cit69] Holton J. L., Beesley C., Jackson M., Venner K., Bhardwaj N., Winchester B., Al-Memar A. (2006). Neuropathol. Appl. Neurobiol..

