# Supplementary material for: Selective and sensitive visualization of endogenous nitric oxide in living cells and animals by a Si-rhodamine deoxylactam-based near-infrared fluorescent probe
Source: Chem Sci. 2017 Aug 2;8(10):6857–64. doi: 10.1039/c7sc02608k (PMC5848605; doi:10.1039/c7sc02608k)
Supplement: Supplementary file 1 [file SC-008-C7SC02608K-s001.pdf]

Electronic Supplementary Material (ESI) for Chemical Science.
This journal is © The Royal Society of Chemistry 2017

Supporting Information

Selective and Sensitive Visualization of Endogenous Nitric Oxide in Living Cells and Animals by a Si-Rhodamine Deoxylactam-Based Near-Infrared Fluorescent Probe

Yingying Huo,[a] Junfeng Miao,[a] Lingjun Han,[c] Yaping Li,[a] Zhe Li,[a] Yawei Shi,[b] and Wei Guo*[a]

[a]*School of Chemistry and Chemical Engineering, Shanxi University, Taiyuan 030006, China.*

[b]*Institute of Biotechnology, Shanxi University, Taiyuan 030006, China.*

[c]*Department of Chemistry, Taiyuan Normal University, Jinzhong 030619, China*

**Corresponding Author E-mail: guow@sxu.edu.cn*

1. General information and methods

All reagents and solvents were purchased from commercial sources and were of the highest grade. Solvents were dried according to standard procedures. All reactions were magnetically stirred and monitored by thin-layer chromatography (TLC). Flash chromatography (FC) was performed using silica gel 60 (200–300 mesh). Absorption spectra were taken on Varian Carry 4000 spectrophotometer. Fluorescence spectra were taken on Hitachi F-7000 fluorescence spectrometer. The ^{1}H NMR and ^{13}C NMR spectra were taken on a Bruker spectrometer, and recorded at 600 and 150 MHz, respectively. The following abbreviations were used to explain the multiplicities: s = singlet; d = doublet; t = triplet; q = quartet; m = multiplet; br = broad. High resolution mass spectra were obtained on a Varian QFT-ESI mass spectrometer. The imaging assays of cells were performed in Zeiss LSM 880+Airyscan Laser Scanning Confocal Microscope, unless otherwise mentioned. The imaging assays of living body were performed in Bruker In-Vivo FX Pro small animal optical imaging system with an excitation filter 620 nm and an emission filter 670 nm.

2. Synthesis

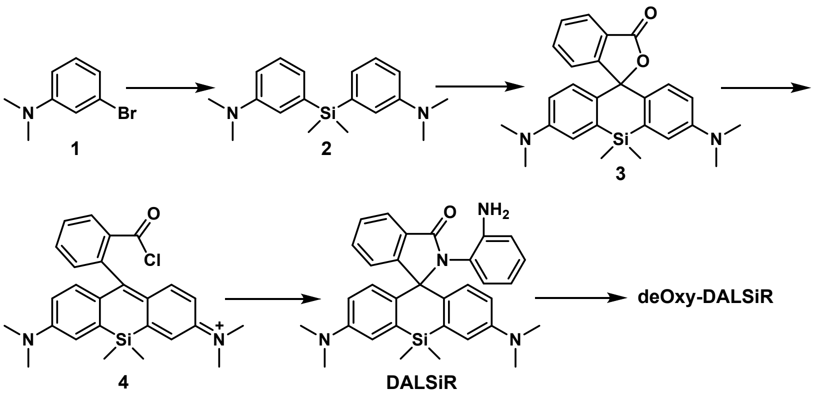

Compound 2: To a 250 mL well-dried flask flushed with nitrogen, 3-bromo-N,N-dimethyaniline **2** (6 g, 30.0 mmol) and diethyl ether (60 mL) were added. After the solution was cooled to 0 °C, n-BuLi (2.4 M in n-hexane, 13.1 mL, 31.5 mmol) was added and the reaction mixture was stirred at 0 °C for 2 h. Dichlorodimethylsilane (2.2 mL, 18.0 mmol) dissolved in diethyl ether (10 mL)

was then added dropwise, and the reaction mixture was slowly warmed to room temperature, then stirred overnight. The reaction was quenched with water (50 mL) and extracted with diethyl ether (50 mL × 3). The organic layers were combined, washed with brine, and dried over anhydrous Na_2SO_4. After filtration and removal of the solvent under reduced pressure, the residue was purified by column chromatography on silica gel (petroleum ether: ethyl acetate = 80 : 1) to afford compound **2** as light yellow oil (3.35 g, 75% yield). ^{1}H NMR (600 MHz, $CDCl_3$) δ 7.27 – 7.24 (m, 2H), 6.96 (s, 2H), 6.95 – 6.91 (m, 2H), 6.81 – 6.76 (m, 2H), 2.94 (s, 12H), 0.55 (s, 6H).

Compound 3: To a 15 mL sealable pressure tube charged with a magnetic stir bar were added compound **2** (1.49g, 5 mmol), 2-formylbenzoic acid (3.75 g, 25 mmol) and copper(II) bromide (112 mg, 0.5 mmol). The tube was sealed tightly and heated at 140 °C for 5 h. After cooling to room temperature, the reaction mixture was dissolved in dichloromethane and washed with 2M NaOH aqueous solution to remove unreacted 2-formylbenzoic acid and other acid side products. The organic layer was washed with brine and dried over anhydrous Na_2SO_4. After filtration and removal of the solvent under reduced pressure, the residue was purified by column chromatography on silica gel (petroleum ether : ethyl acetate : triethylamine = 50 : 1 : 1). And the product was recrystallized from dichloromethane/petroleum ether to give compound **3** as colorless needle crystals(0.97 g，45%).^{1}H NMR (600 MHz, CDCl3) δ 7.96 (d, J = 7.7 Hz, 1H), 7.63 (td, J = 7.5, 1.0 Hz, 1H), 7.55 – 7.52 (m, 1H), 7.30 (d, J = 7.7 Hz, 1H), 6.98 (d, J = 2.1 Hz, 2H), 6.79 (d, J = 8.9 Hz, 2H), 6.56 (dd, J = 8.9, 2.7 Hz, 2H), 2.96 (s, 12H), 0.64 (s, 3H), 0.61 (s, 3H); ^{13}C NMR (151 MHz, CDCl3) δ 170.71, 154.40, 150.02, 149.25, 137.00, 134.07, 133.66, 129.10, 128.75,128.68，128.17, 127.03, 125.65, 125.43, 124.58, 123.06, 116.65, 113.36, 112.26, 83.51, 40.32, 40.36, 0.44, -1.50; ESI-MS: Calcd for $[M+H]^+$ 429.1993, Found 429.1992.

Compound DALSiR: To a stirred solution of compound **3** (428 mg, 1.0 mmol) in dry 2−dichloroethane (5.0 mL) at room temperature, phosphorus oxychloride (460 mg, 3.0 mmol) was added dropwise over a period of 5 minutes. After being refluxed for 4

hours, the reaction mixture was cooled and concentrated under vacuum to give a blue solid (**4**). This blue solid was dissolved in dry acetonitrile (5.0 mL), which was then slowly added to a solution of *o*–diaminobenzene (540 mg, 5.0 mmol) in dry acetonitrile (5.0 mL) containing triethylamine (5.0 mL). After stirred at room temperature overnight, the mixture was concentrated under vacuum and the crude product was purified by column chromatography on silica gel (ethyl acetate: petroleum ether = 1 : 4) to give **DALSiR** as a white solid (327 mg, 57% yield). ^{1}H NMR (600 MHz, $CDCl_3$) δ 8.06 (dd, J = 5.4, 3.1 Hz, 1H), 7.55 (dd, J = 5.6, 3.1 Hz, 2H), 7.14 (dd, J = 5.3, 3.1 Hz, 1H), 6.92 (t, J = 7.6 Hz, 1H), 6.79 – 6.73 (m, 4H), 6.62 (dd, J = 9.0, 2.7 Hz, 2H), 6.53 (d, J = 8.0 Hz, 1H), 6.36 (t, J = 7.6 Hz, 1H), 5.85 (d, J = 7.9 Hz, 1H), 3.24 (br, 2H), 2.98 (s, 12H), 0.47 (s, 3H), -0.28 (s, 3H); ^{13}C NMR (151 MHz, $CDCl_3$) δ 167.46, 155.33, 148.70, 145.33, 137.15, 132.74, 132.38, 132.12, 130.30, 130.10, 128.81, 127.88, 124.09, 123.54, 122.07, 118.17, 116.79, 115.29, 114.45, 40.23, -0.53, -1.46; ESI-MS: Calcd for $[M+Na]^+$ 541.2394, Found 541.2396.

deOxy-DALSiR: To a stirred solution of compound **DALSiR** (103 mg, 0.2 mmol) in dry THF (5 ml) at room temperature, the solution of BH_3 in THF (1 M, 1.1 mmol) was added dropwise under N_2 atmosphere. After being refluxed for 12 hours, the reaction mixture was cooled and cautiously quenched by addition of methanol (5 mL). The resulting mixture was concentrated under vacuum and the crude product was purified by column chromatography on silica gel (ethyl acetate: petroleum ether = 1 : 5) to give **deOxy-DALSiR** as a blue solid (53 mg, 53% yield). ^{1}H NMR (600 MHz, CD_3CN) δ 7.47 (d, J = 7.5 Hz, 1H), 7.36 (t, J = 7.5 Hz, 1H), 7.27 (t, J = 7.4 Hz, 1H), 6.86 (d, J = 9.0 Hz, 2H), 6.82 (d, J = 2.5 Hz, 2H), 6.75 – 6.66 (m, 4H), 6.45 (d, J = 7.9 Hz, 1H), 6.16 (t, J = 7.6 Hz, 1H), 5.99 (d, J = 8.0 Hz, 1H), 4.59 (s, 2H), 3.60 (br, 2H), 2.93 (s, 12H), 0.40 (s, 3H), -0.26 (s, 3H); ^{13}C NMR (151 MHz, CD_3CN) δ 151.89, 148.49, 147.01, 139.98, 138.36, 138.75, 131.72, 131.36, 129.08, 127.55, 126.60, 125.54, 124.26, 121.87, 116.66, 115.26, 115.03, 114.39, 39.66, 26.62, -1.39, -1.95; ESI-MS: Calcd for $[M+H]^+$ 505.2782, Found 505.2783.

deOxy-DALSiR-T: To a stirred solution of **deOxy-DALSiR** (50 mg, 0.1 mmol) in

AcOH (0.5 mL) and methanol (5 mL), $NaNO_2$ (21 mg, 0.3 mmol) was added at room temperature. Stirring was continued for 3 h. The mixture was concentrated under vacuum, and the crude product was purified by column chromatography on silica gel (methanol: dichloromethane = 1 : 8) to give **deOxy-DALSiR-T** as a blue solid (44 mg, 80% yield). ^{1}H NMR (600 MHz, MeOD) δ 7.99 (d, J = 7.7 Hz, 1H), 7.75 (t, J = 7.5 Hz, 1H), 7.64 (t, J = 7.8 Hz, 2H), 7.37 – 7.34 (m, 1H), 7.31 (t, J = 7.4 Hz, 1H), 7.26 (d, J = 8.2 Hz, 1H), 7.23 – 7.19 (m, 3H), 6.64 (d, J = 9.6 Hz, 2H), 6.32 (dd, J = 9.6, 2.7 Hz, 2H), 5.69 (s, 2H), 3.31 (s, 12H), 0.79 (s, 3H), 0.48 (s, 3H).^{13}C NMR (151 MHz, MeOD) δ 165.81, 154.05, 148.09, 145.36, 139.70, 139.45, 132.79, 132.70, 131.28, 129.97, 129.17, 128.69, 127.27, 126.54, 123.84, 120.58, 118.41, 113.04, 110.28, 50.88, 39.43, -2.03, -2.71; ESI-MS: Calcd for $[M+H]^+$ 516.2578, Found 516.2579.

Compound DALR: The **c**ompound was synthesized according to the reported method.[1] ^{1}H NMR (600 MHz, CDCl3) δ 8.04 (dd, J = 6.4, 1.7 Hz, 1H), 7.62 – 7.53 (m, 2H), 7.28 – 7.24 (m, 1H), 6.97 (td, J = 8.0, 1.4 Hz, 1H), 6.66 (d, J = 8.8 Hz, 2H), 6.57 (dd, J = 8.0, 1.0 Hz, 1H), 6.46 – 6.41 (m, 1H), 6.34 (d, J = 6.2 Hz, 2H), 6.28 (s, 2H), 6.12 (dd, J = 7.9, 1.1 Hz, 1H), 3.38 – 3.29 (m, 8H), 1.17 (t, J = 7.0 Hz, 12H). ^{13}C NMR (151 MHz, CDCl3) δ 166.44, 153.98, 152.40, 148.91, 144.52, 132.63, 131.96, 128.83, 128.75, 128.67, 128.37, 124.30, 123.46, 122.18, 118.22, 117.01, 107.98, 106.96, 98.03, 68.06, 44.40, 12.53; ESI-MS: Calcd for $[M+H]^+$ 533.2911, Found 533.2912.

3. Preparation of the test solution

Stock solution of **deOxy-DALSiR** in CH_3CN (2 mM) was used to prepare the working solutions in PBS (50 mM, pH 7.4, containing 20% CH_3CN) with a final concentration of 4.0 μM. For assays in chemical system, the NO stock solution in deionized water was used, which was prepared by bubbling NO gas into a NaOH solution to eliminate NO_2 generated from the reaction of NO and O_2, and then into deoxygenated deionized water for 30 min. The concentration of the resulting NO stock solution was determined to be 1.8 mM by Griess method. For assays in cells, a commercially available NO donor NOC-9 (dissolved in 0.1 M NaOH solution) was

used. For assays in chemical system, $ONOO^-$ solution, which was synthesized according to a reported procedure,[2] was used, and its concentration was determined using an extinction coefficient of 1670 $M^{-1}cm^{-1}$ at 302 nm. For cell and living animal imaging assays, $ONOO^-$ was generated from a commercially available $ONOO^-$ donor SIN-1 (dissolved in 0.1 M NaOH solution). $O_2^{\bullet -}$ was prepared by adding KO_2 (7.1 mg) and 18-Crown-6 (1 equiv) to dry dimethyl sulfoxide (5 mL) and stirring vigorously for 10 min. $HO^{\bullet}$ was generated *in situ* by the Fenton reaction, and its concentration was equal to the Fe(II) concentration. $^1O_2$ was generated *in situ* by adding NaClO solution into H_2O_2 solution (10 eq), and its concentration was equal to the NaClO concentration. H_2O_2 solution was prepared by dilution of commercial H_2O_2 solution in deionized water, and its concentration was determined by using an extinction coefficient of 43.6 $M^{-1}cm^{-1}$ at 240 nm. NaClO solution was prepared by the dilution of commercial NaClO solution in deionized water, and its concentration was determined using an extinction coefficient of 350 $M^{-1}cm^{-1}$ at 292 nm. The aqueous solutions of $NaNO_2$ was freshly prepared and used as NO_2^- source. The aqueous solutions of K^+, Ca^{2+}, Na^+, Mg^{2+}, Al^{3+}, Zn^{2+}, Fe^{2+}, Fe^{3+}, Cu^+, and Cu^{2+} were freshly prepared from their chloride salts. The aqueous solutions of Cys/GSH and the DMSO solutions of DHA/AA/MGO were freshly prepared. For spectra studies, various analytes, except $^{\bullet}OH$ and $^1O_2$, were directly added to the solution of **deOxy-DALSiR** (4 μM) in PBS (50 mM, pH 7.4, containing 20% CH_3CN), and then fluorescence spectra were recorded in the indicated time points. For $^{\bullet}OH$ or $^1O_2$, **deOxy-DALSiR** and H_2O_2 were premixed, and then Fe^{2+} or ClO^- was added to the mixture.

4. Cell culture and fluorescence imaging

4.1 Cell culture

The HeLa cell line, Raw 264.7 macrophage cell line, EA.hy 926 cell line, and pancreatic β-cell (INS-1) line were kindly provided by Key Laboratory of Chemical Biology and Molecular Engineering of Ministry of Education (China). Cells were grown in Dulbecco's modification of Eagle's medium (DMEM) supplemented with 10 % FBS (Fetal Bovine Serum) and 1% antibiotics at 37 ºC in humidified environment of

5% CO_2. Cells were plated on glass bottom cell culture dish (30 mm) and allowed to adhere for 12 hours. Before experiments, cells were washed with phosphate buffer saline (PBS) 3 times.

4.2 Imaging exogenous and endogenous NO in HeLa cells, RAW264.7 macrophages, and pancreatic β-cells (INS-1)

To test the selectivity of **deOxy-DALSiR** for NO in cell environment, HeLa cells were pretreated with **deOxy-DALSiR** (2 μM) in PBS for 20 min, and then treated with NOC-9 and representative ROS, such as H_2O_2, ClO^-, SIN-1 (a commercially available $ONOO^-$ donor), respectively, for 20 min. For imaging of intracellular basal NO, Raw 264.7 cells or INS-1 cells were directly treated with **deOxy-DALSiR** (2 μM, 20 min). For imaging of stimulator-induced NO, Raw 264.7 cells (or INS-1 cells) were pretreated with stimulator LPS (20 μg/mL, 6 h)/INF-γ (150 units/mL, 6 h) [or STZ (0.5 mM, 12 h)], and then treated with **deOxy-DALSiR** (2 μM, 20 min). For inhibition assays, Raw 264.7 cells (or INS-1 cells) were pretreated with AG (0.5 mM) in the presence of LPS (20 μg/mL, 6 h)/INF-γ (150 units/mL, 6 h) [or STZ (0.5 mM, 12 h)], and then treated with **deOxy-DALSiR** (2 μM, 20 min). After each treatment, the cells were washed with PBS 3 times. Emission was collected at 650−750 nm (λ_{ex}: 633 nm).

4.3 Imaging endogenous NO in EA.hy926 endothelial cells after oxygen glucose deprivation (OGD)

To mimic the ischemia *in vitro*, EA.hy926 endothelial cells were subjected to OGD condition. Briefly, the airtight hypoxia chamber was flushed with 95% N_2/5% CO_2 for 1 h before experiments. To obtain OGD, the standard culture medium was replaced with glucose-free Hank's balanced salt solution (HBSS), and then probe **deOxy-DALSiR** (2 μM) was added into the media; after that, the **deOxy-DALSiR**-containing cultures were placed in hypoxia chamber flushed with 95% N_2/5% CO_2 and maintained at 37 °C. Following 0.5 h, 1 h, and 2 h of OGD, respectively, the cells were then removed from hypoxia chamber, and images were obtained in a EVOS FL

Auto imaging system. The excitation and emission bandpasses of the standard Cy5 filter set were used.

4.4 *Cell costaining studies*.

To evaluate the subcellular localization of **deOxy-DALSiR**, HeLa cells were incubated with **deOxy-DALSiR** (2.0 μM) and MitoTracker green FM (0.2 μM) (or LysoTracker green DN-26 (0.07 μM)) in DMEM for 20 min. After washing with PBS 3 times, the cells in PBS were treated with NOC-9 (20 μM) to light up the probe. For **deOxy-DALSiR**, emission was collected at 650–750 nm (λ_{ex} = 633 nm). For MitoTracker green FM or LysoTracker green DN-26, emission was collected at 500-600 nm (λ_{ex} = 488 nm).

2.6. Photostability test

The photostability of **deOxy-DALSiR** in the absence and presence of NOC-9 was tested in HeLa cells. The cells were plated on glass bottom cell culture dish (30 mm) and allowed to adhere for 12 hours. After washed with PBS 3 times, the cells were treated with 2 μM **deOxy-DALSiR** for 20 min in DMEM. The **deOxy-DALSiR**-loaded cells were then continuously irradiated by semiconductor laser under Zeiss LSM 880+Airyscan Laser Scanning Confocal Microscope for 60 min, and the representative images were obatined in different time points. Similarly, the cells pre-treated with **deOxy-DALSiR** (2 μM, 20 min) and then treated with NOC-9 (20 μM, 20 min) were also subjected to the same irradiation treatment. Emission was collected at 650–750 nm (λ_{ex} = 633 nm).

5. MTT assays

HeLa Cells were seeded in 96-well microplates in DMEM medium supplemented with 10 % FBS (Fetal Bovine Serum) and 1% antibiotics at 37 ºC in humidified environment of 5% CO_2. After 24 h of cell attachment, the plates were washed with PBS, followed by addition of increasing concentrations of **deOxy-DALSiR** or **deOxy-DALSiR-T** (2−50 μM) in DMEM. The cells were then incubated at 37 ºC in

an atmosphere of 5% CO_2 and 95% air for 24 h, followed by standard MTT assays (n= 6). Untreated assays (n = 6) were also conducted under the same conditions.

6. Imaging endogenous NO in LPS or STZ-treated mouse models

A Bruker In-Vivo FX Pro small animal optical imaging system with an excitation filter 620 nm and an emission filter 670 nm was used for the living animal imaging assays. The mouse was first i.p. injected with LPS (1 mg/ml, 100 μL) or STZ (40 mg/kg), and then **deOxy-DALSiR** (2 μM, 100 μL) after 24 h. After 30 min, the mouse was anesthetized and applied to the imaging system. The untreated mouse was used as a control. The experiments were performed in compliance with the relevant laws and institutional guidelines, and animal care and handing procedures were reviewed and approved by Animal Care and Use Committee of Shanxi University. Informed consent was obtained for any experimentation with human subjects.

7. Supplementary Spectra

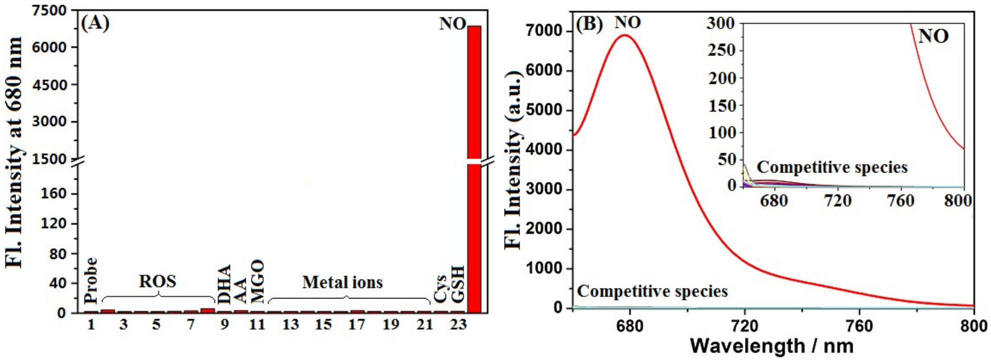

Figure S1 (A) Fluorescence intensities of **deOxy-DALSiR** (4 μM) treated with various biologically relevant species for 5 min. (1) **deOxy-DALSiR** only; (2) HClO; (3) H_2O_2; (4) $^1O_2$; (5) $O_2^{\bullet -}$; (6) $^{\bullet}OH$; (7) NO_2^-; (8) $ONOO^-$; (9) DHA; (10) AA; (11) MGO; (12) K^+; (13) Ca^{2+}; (14) Na^+; (15) Mg^{2+}; (16) Al^{3+}; (17) Zn^{2+}; (18) Fe^{2+}; (19) Fe^{3+}; (20) Cu^+; (21) Cu^{2+}; (22) Cys; (23) GSH; (24) NO. Concentrations for (2–8), 100 μM; for (9–11), 1 mM; for (12–21), 100 μM; for (22), 200 μM; for (23) 1 mM; for (24), 30 μM. (B) The corresponding fluorescence spectra. Conditions: PBS buffer (50 mM, pH 7.4, containing 20% CH_3CN); T = 25 ^oC; λ_{ex} = 645 nm; λ_{em} = 680 nm; Slits: 5/10 nm.

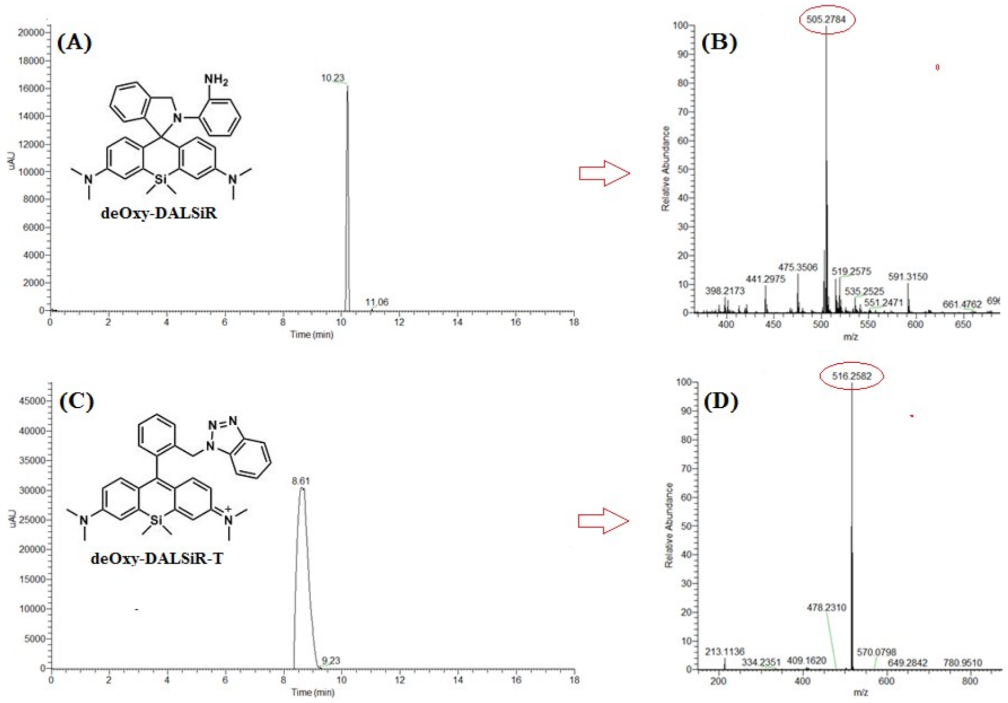

Figure S2 HPLC-MS results of **deOxy-DALSiR** in the absence (A,B) and presence (C,D) of excessive NO in PBS buffer (50 mM, pH 7.4, containing 20% CH_3CN).

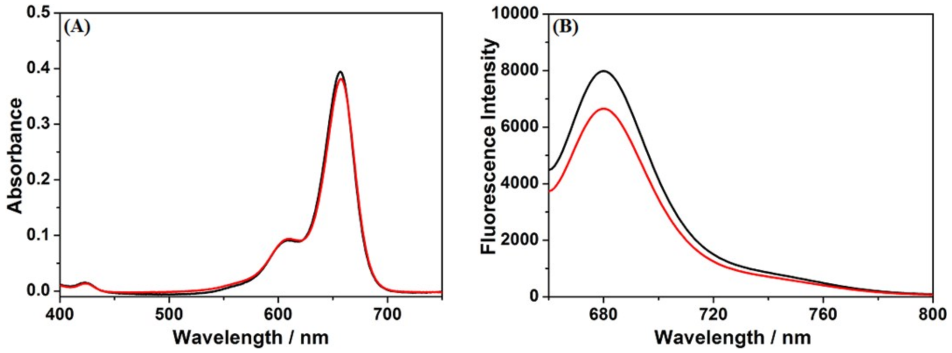

Figure S3 (A) Absorption spectra of **deOxy-DALSiR-T** (black) and **deOxy-DALSiR** treated with NO (red), respectively. (B) Fluorescence spectra of **deOxy-DALSiR-T** (black) and **deOxy-DALSiR** treated with NO (red), respectively. Conditions: PBS buffer (50 mM, pH 7.4, containing 20% CH_3CN); T = 25 ^{o}C; λ_{ex} = 645 nm; λ_{em} = 680 nm; Slits: 5/10 nm; voltage: 600 V.

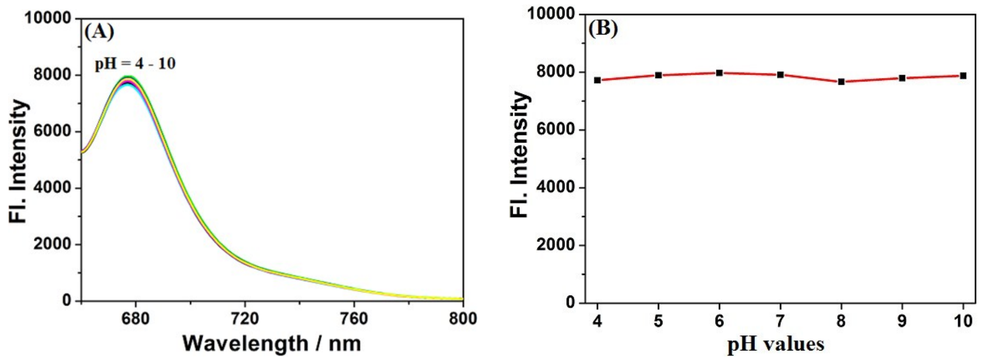

Figure S4 (A) Effects of pH on fluorescence spectra of **deOxy-DALSiR-T**. (B) The corresponding fluorescence intensities at 680 nm. Condition: B-R buffer (20 mM, pH = 4–10, containing 20% CH_3CN). λ_{ex} = 645 nm; λ_{em} = 680 nm; Slits: 5/10 nm; voltage: 600 V.

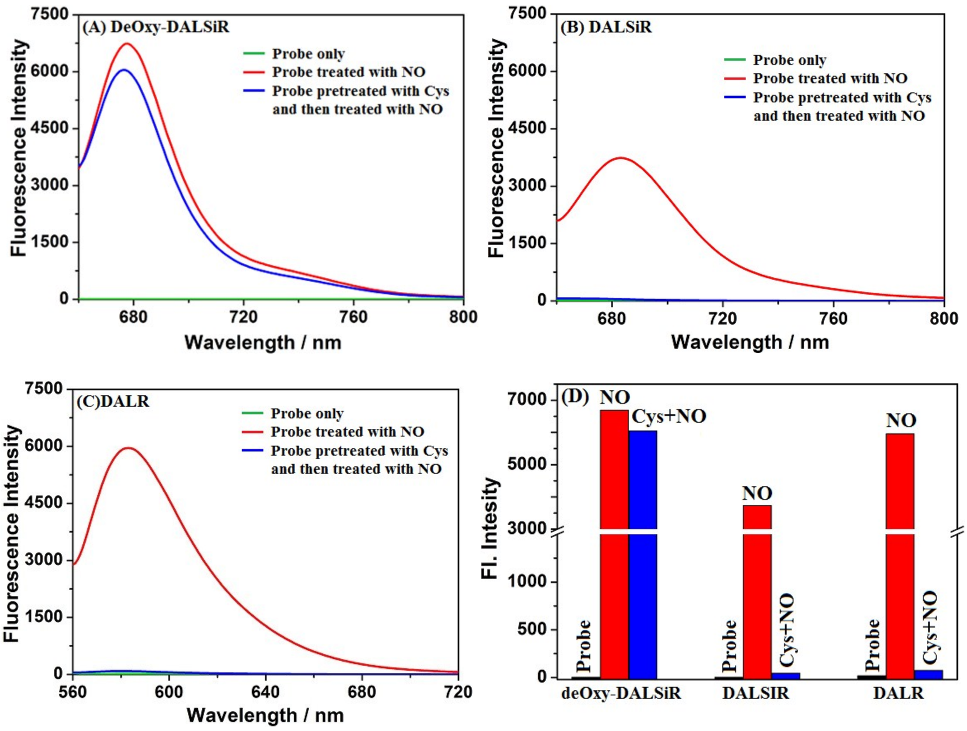

Figure S5 (A-C) Fluorescence spectra of **deOxy-DALSiR**, **DALSiR**, and **DALR** (4 μM for each) treated with NO (80 μM for **deOxy-DALSiR**; 400 μM for **DALSiR** and **DALR**) in the absense and presence of Cys (200 μM). (D) The corresponding bar graphs. Conditions: PBS buffer (50 mM, pH 7.4, containing 20% CH_3CN); T = 25 ^oC; λ_{ex} = 645 nm; λ_{em} = 680 nm; Slits: 5/10 nm; voltage: 600 V.

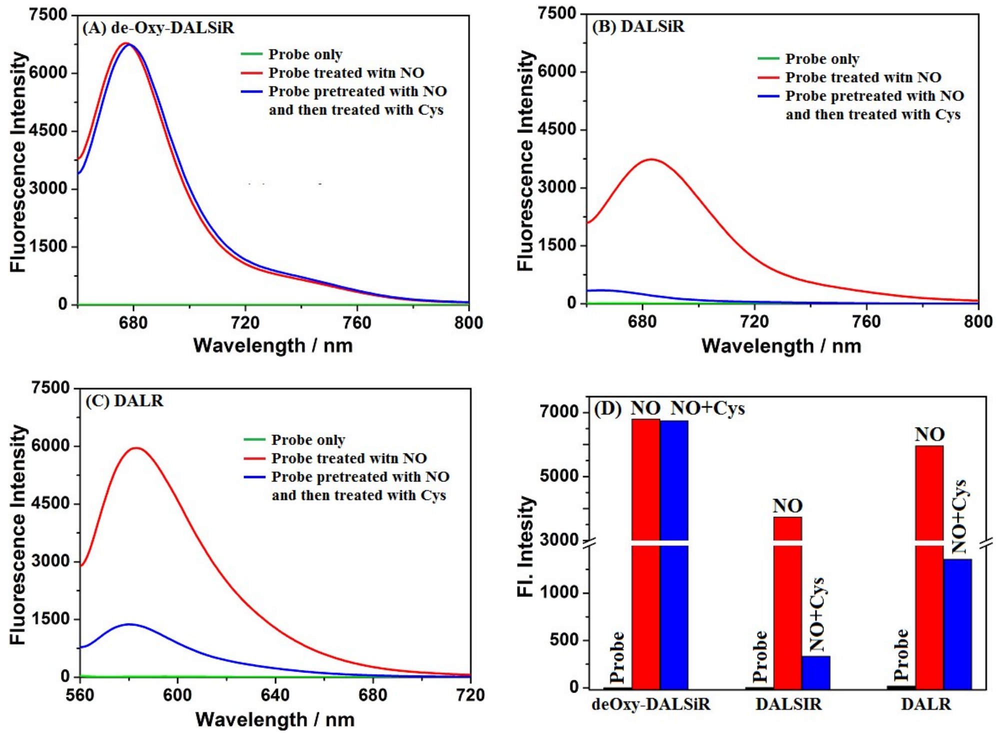

Figure S6 (A-C) Fluorescence spectra of **deOxy-DALSiR**, **DALSiR**, and **DALR** (4 μM for each) pretreated with NO (80 μM for **deOxy-DALSiR**; 400 μM for **DALSiR** and **DALR**) and then treated with Cys (200 μM). (D) The corresponding bar graphs. Conditions: PBS buffer (50 mM, pH 7.4, containing 20% CH_3CN); T = 25 ^oC; λ_{ex} = 645 nm; λ_{em} = 680 nm; Slits: 5/10 nm; voltage: 600 V.

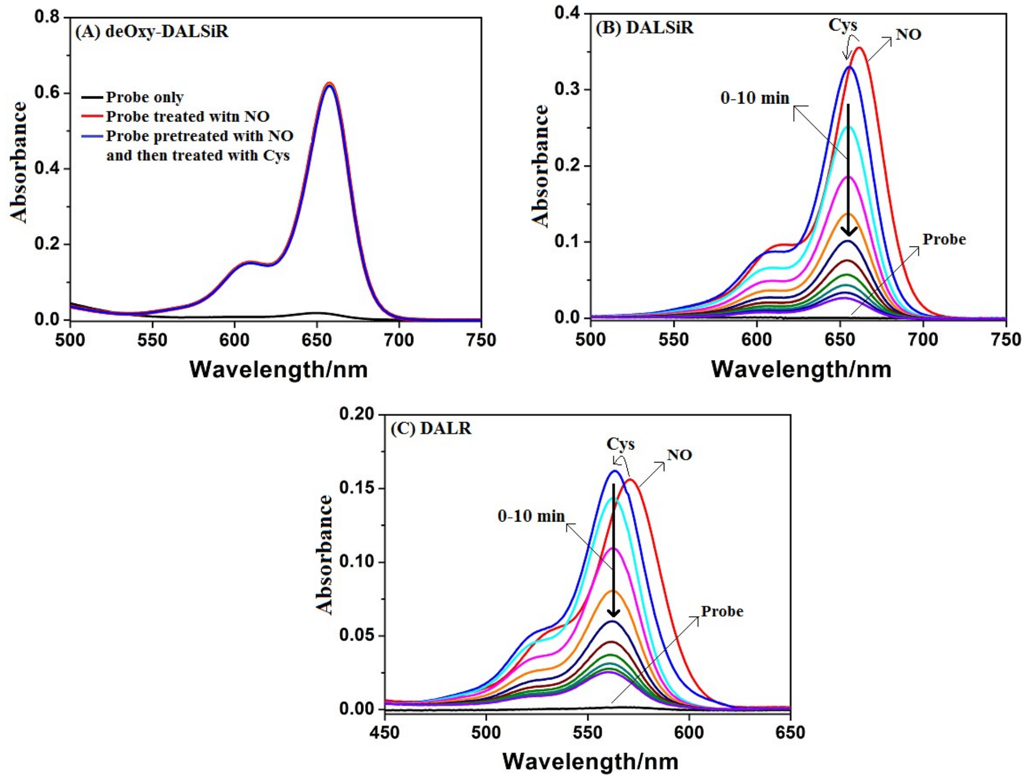

Figure S7 (A-C) Absorption spectra of **deOxy-DALSiR**, **DALSiR**, and **DALR** (4 μM) pretreated with NO (80 μM for **deOxy-DALSiR**; 400 μM for **DALSiR** and **DALR**) for 1 min, and then treated with Cys (200 μM) for 1−10 min. Conditions: PBS buffer (50 mM, pH 7.4, containing 20% CH_3CN); T = 25 °C.

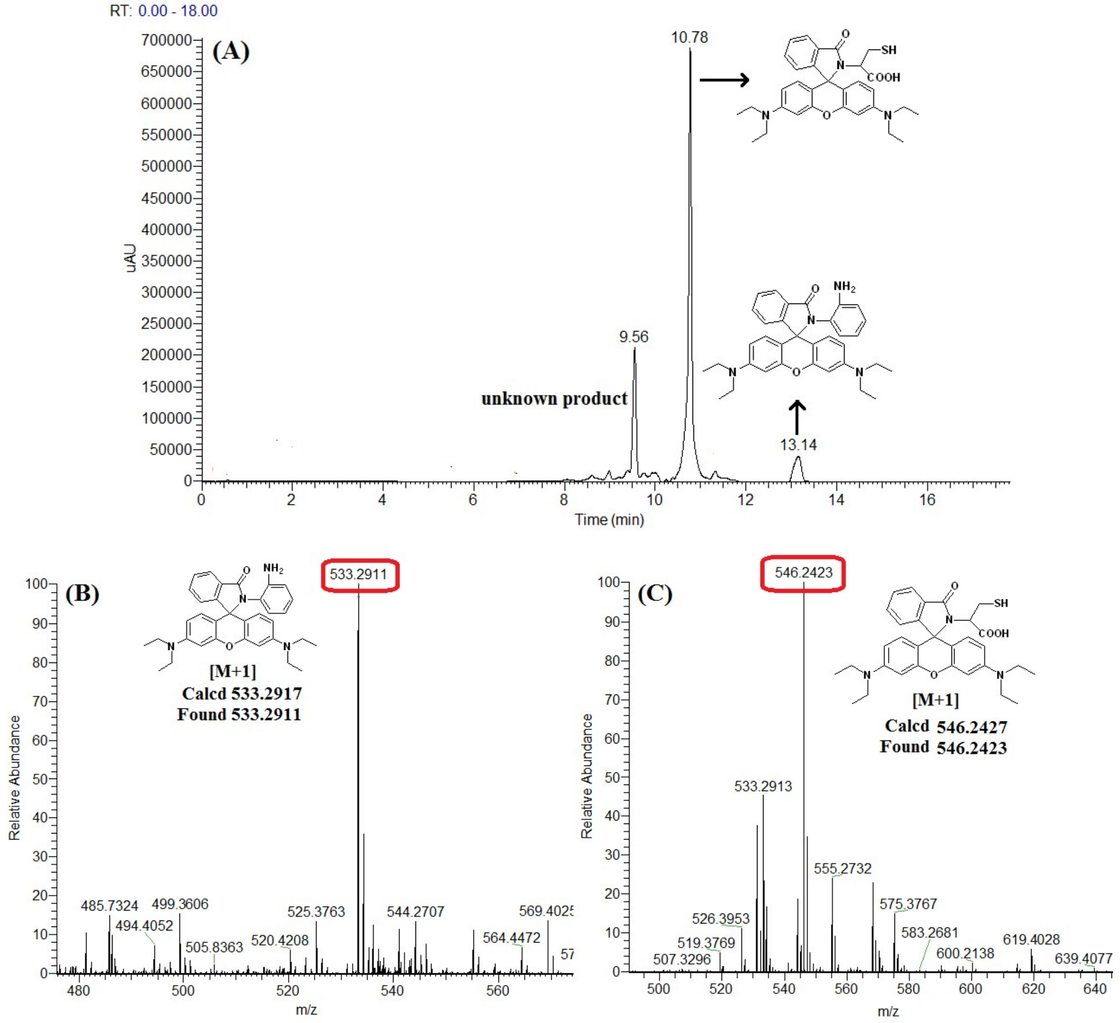

Figure S8 (A) HPLC result of **DALR** pretreated with 100 equiv of NO for 1 min, and then treated with 50 equiv of Cys in PBS buffer (50 mM, pH 7.4, containing 20% CH_3CN). (B) MS chart of the peak at the retention time of 13.14 min in (A). (C) MS chart of the peak at the retention time of 10.78 min in (A).

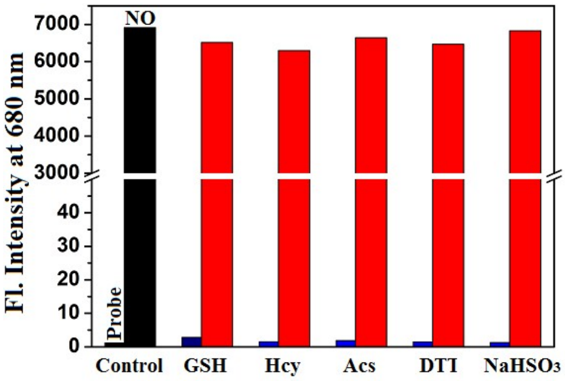

Figure S9 Fluorescence intensities of **deOxy-DALSiR** (4 μM) treated with GSH (1 mM), Hcy (200 μM), ascorbate (Asc, 200 μM), dithiothreitol (DTT, 200 μM), and $NaHSO_3$ (200 μM), respectively (blue bar), and then treated with NO (80 μM) (red bar). Conditions: PBS buffer (50 mM, pH 7.4, containing 20% CH_3CN); T = 25 ^{o}C; λ_{ex} = 645 nm; λ_{em} = 680 nm; Slits: 5/10 nm; voltage: 600 V.

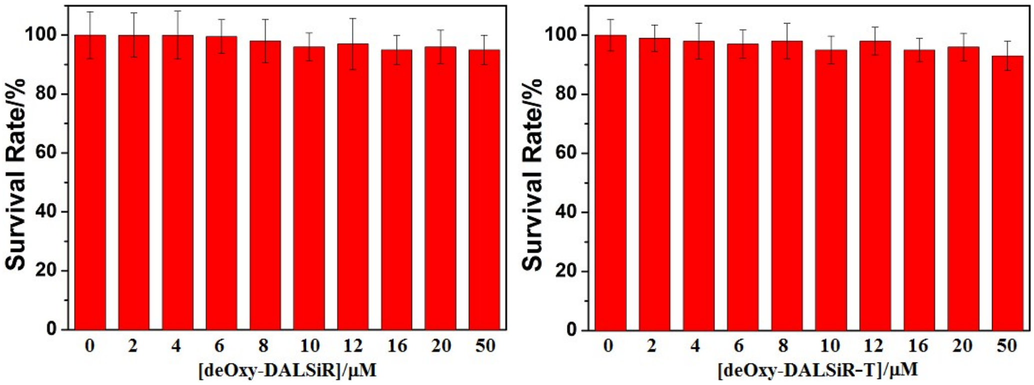

Figure S10 Percentage of viable HeLa cells after treated with increasing concentrations of **deOxy-DALSiR** (left) and **deOxy-DALSiR-T** (right), respectively, for 24 hours.

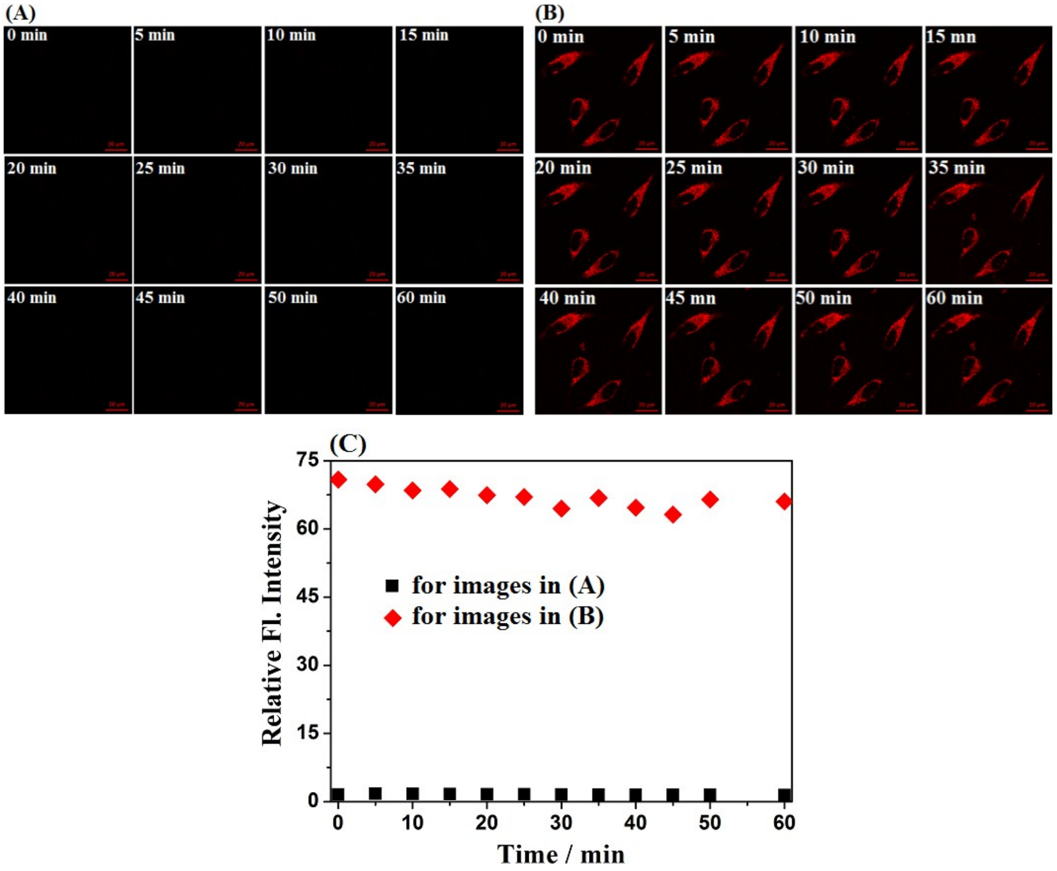

Figure S11 (A, B) Confocal images of the **deOxy-DALSiR** (2 μM)-loaded HeLa cells continuously irradiated by semiconductor laser (633 nm) for 60 min in the absence (A) and presence (B) of NOC-9 (20 μM). The representative images were obatined in the indicated time points. (C) Average fluorescence intensity from images of (A) and (B). Emission was collected at 650−750 nm (λ_{ex}: 633 nm). Scale bar: 20 μm. For details of the photostability assays, see Supplementary Video 1 and Video 2.

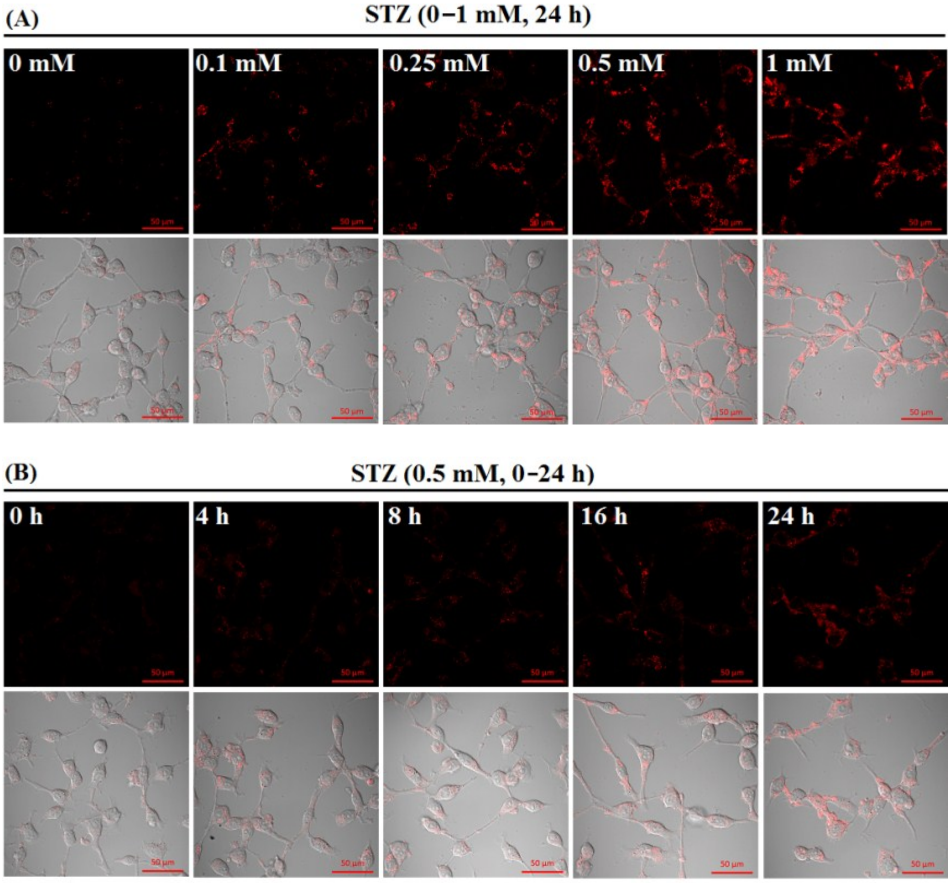

Figure S12 (A) Fluorescence images of pancreatic β-cells (INS-1) pretreated with the increased concentrations of STZ (0−1 mM, 24 h) and then treated with **deOxy-DALSiR** (2 μM, 20 min). (B) Fluorescence images of pancreatic β-cells (INS-1) pretreated with STZ (0.5 mM, 0–24 h) and then treated with **deOxy-DALSiR** (2 μM, 20 min). Emission was collected at 650−750 nm (λ_{ex}: 633 nm).

8. ^{1}H NMR, ^{13}C NMR, and HRMS Charts

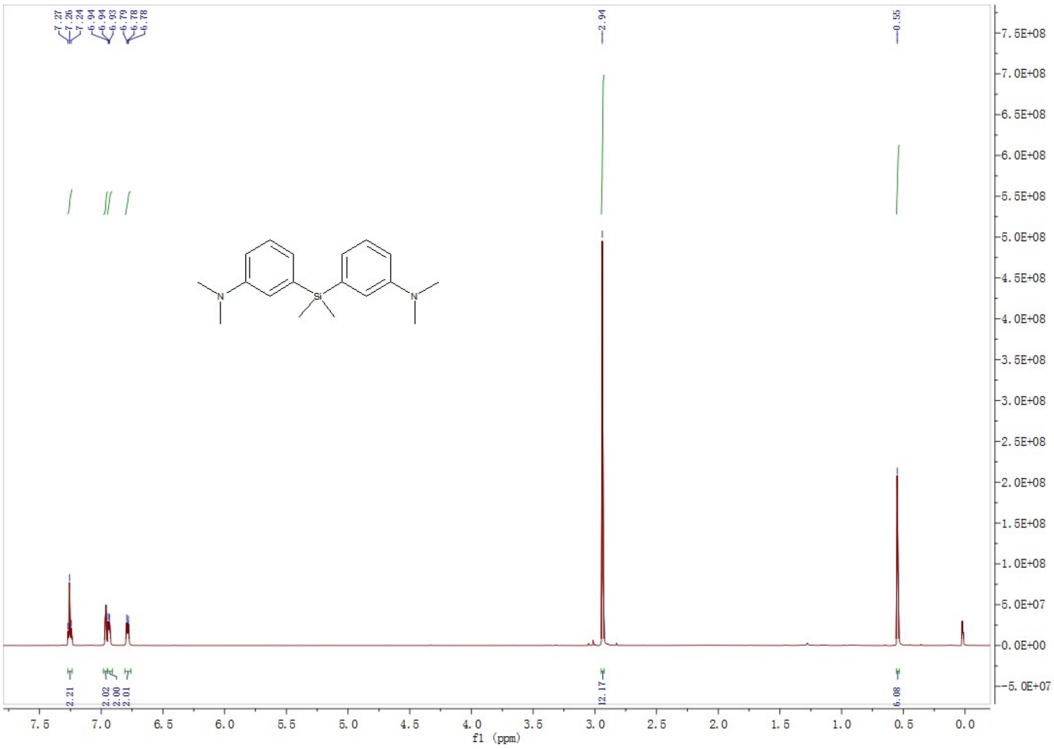

Figure S13 ^{1}H NMR chart of compound **2** ($CDCl_3$, 600 MHz).

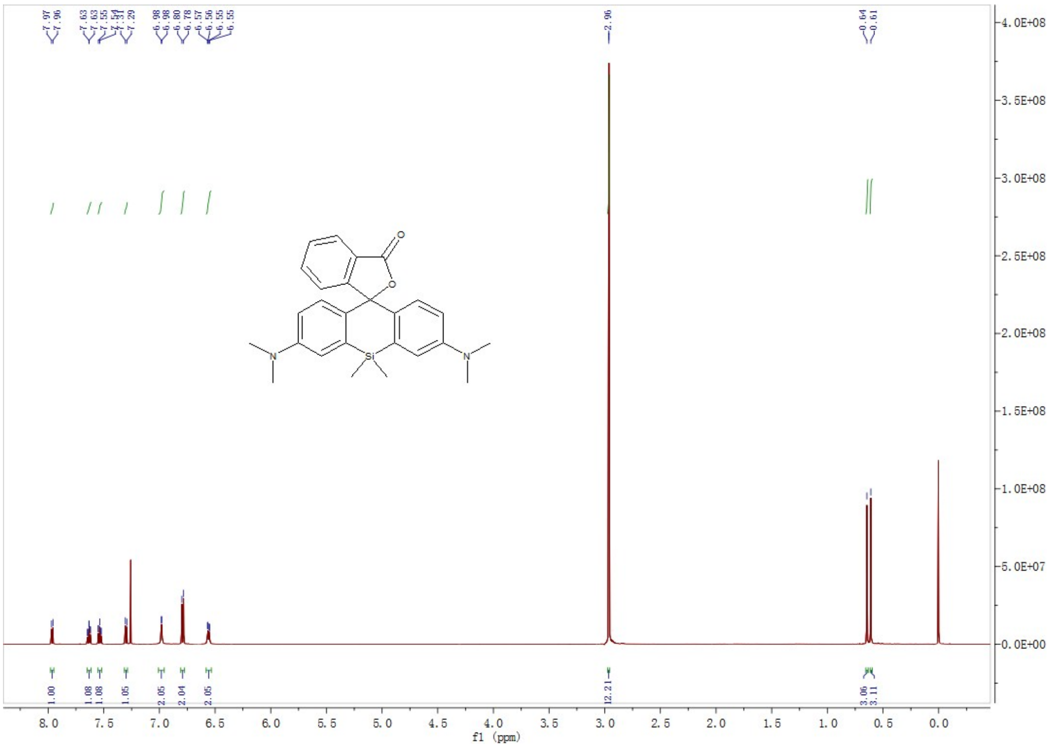

Figure S14 ^{1}H NMR chart of compound **3** ($CDCl_3$, 600 MHz).

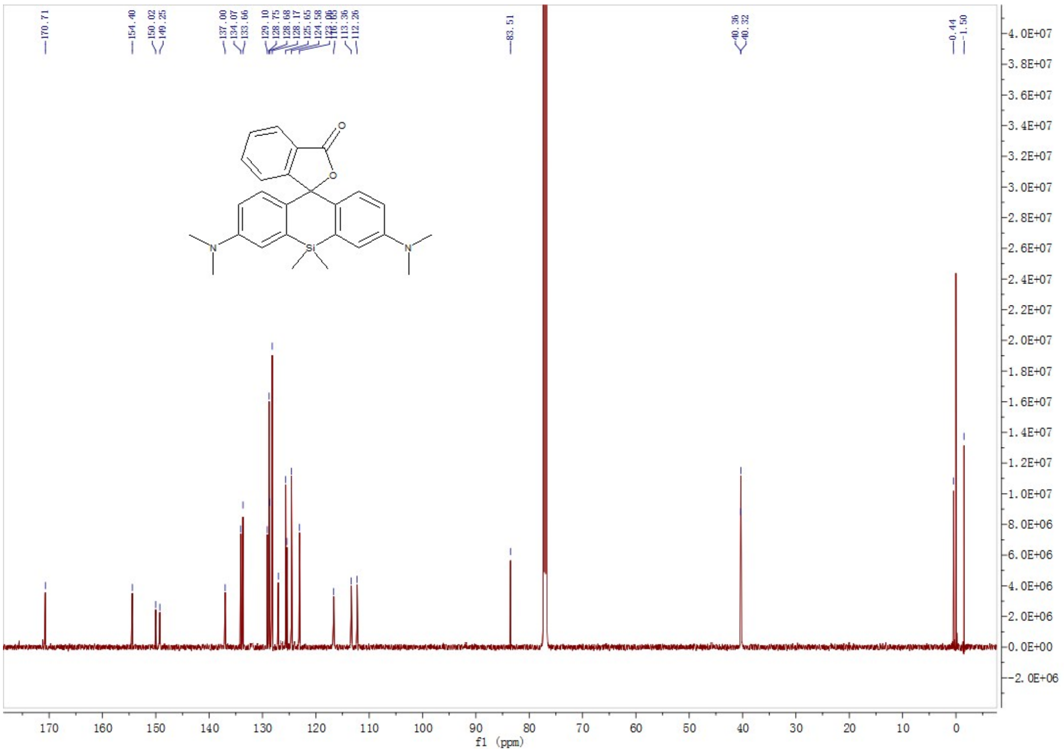

Figure S15 ^{13}C NMR chart of compound **3** ($CDCl_3$, 600 MHz).

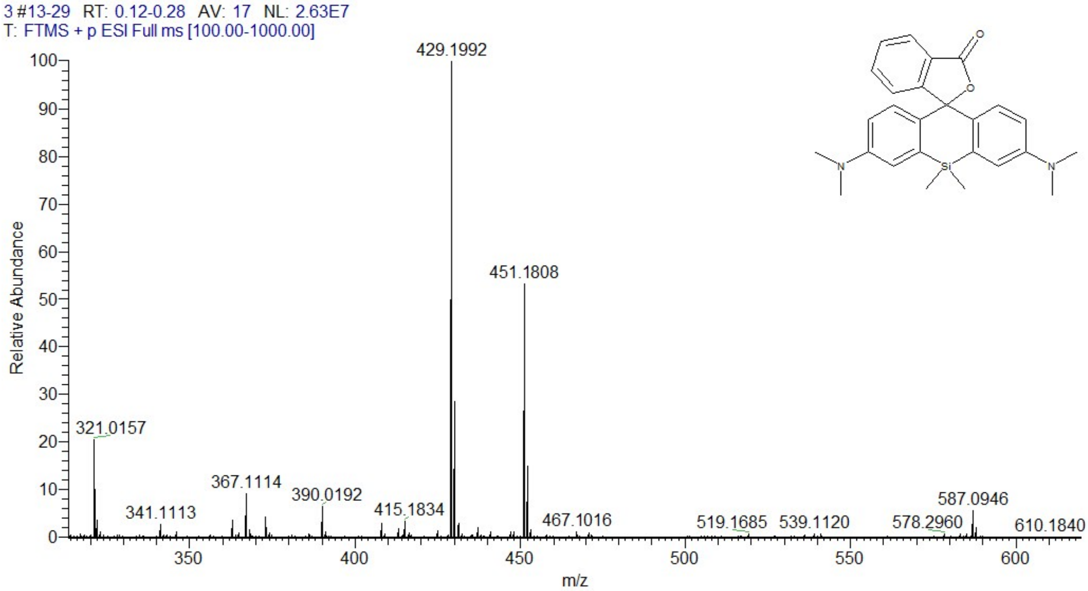

Figure S16 HRMS chart of compound **3**.

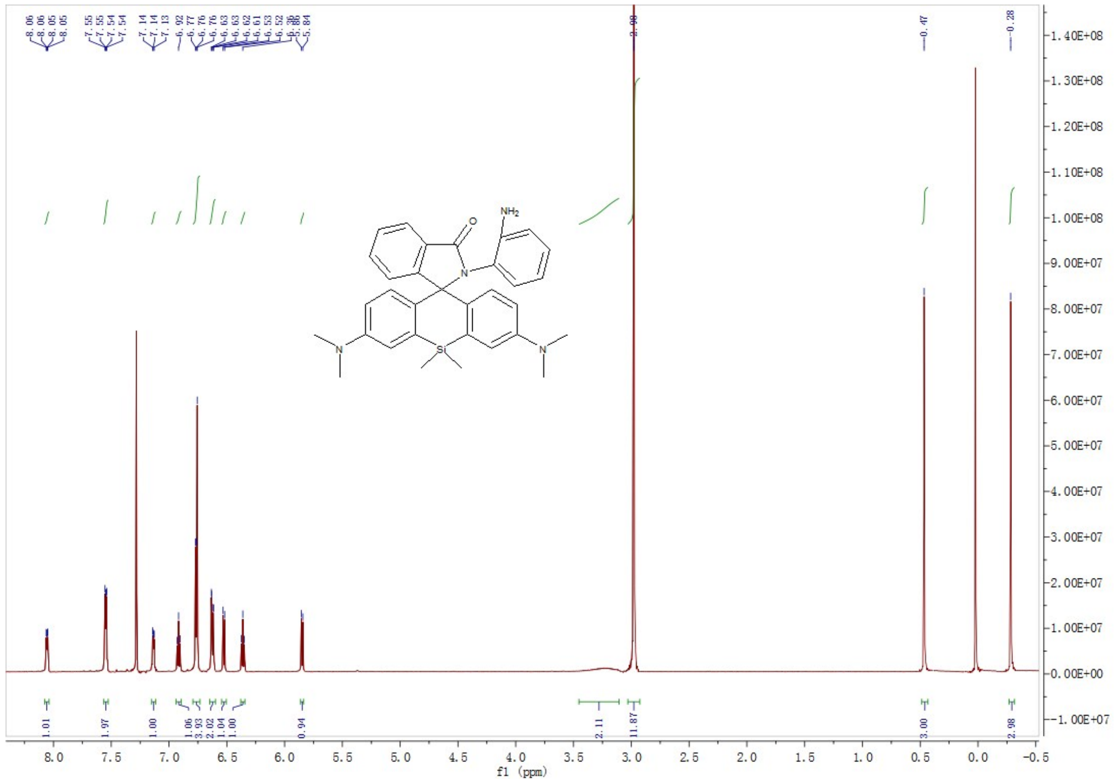

Figure S17 ^{1}H NMR chart of **DALSiR** ($CDCl_3$, 600 MHz).

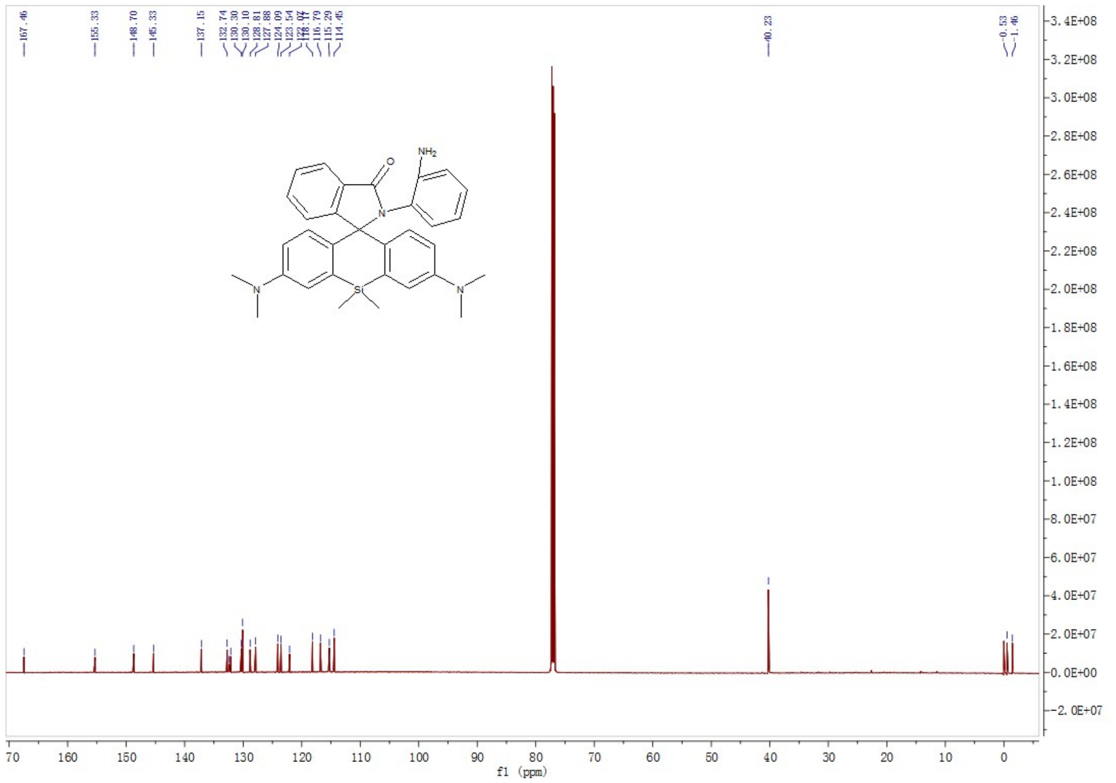

Figure S18 ^{13}C NMR chart of **DALSiR** ($CDCl_3$, 600 MHz).

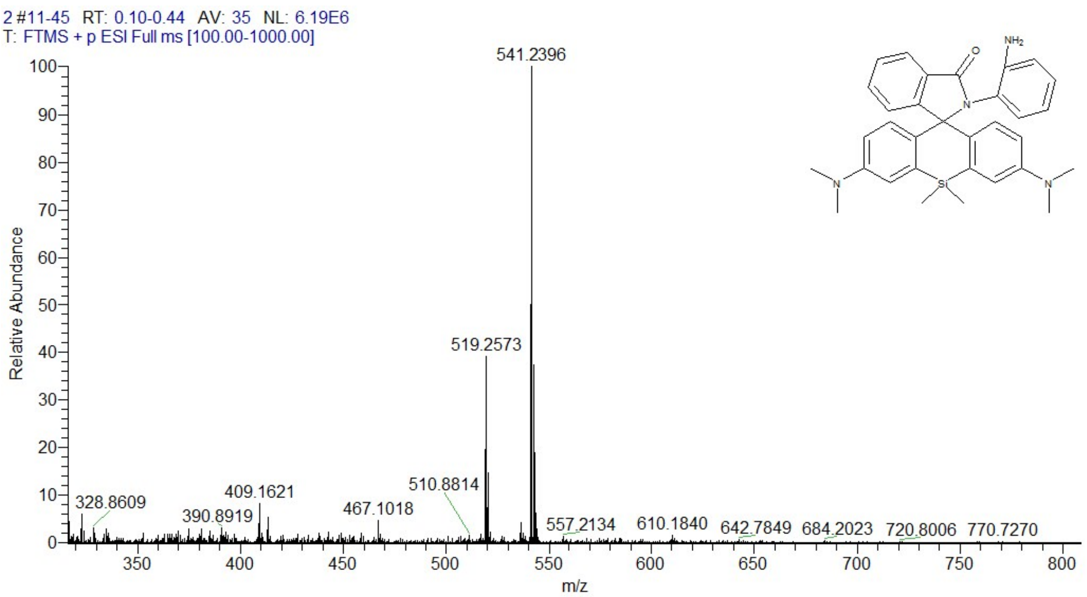

Figure S19 HRMS chart of **DALSiR**.

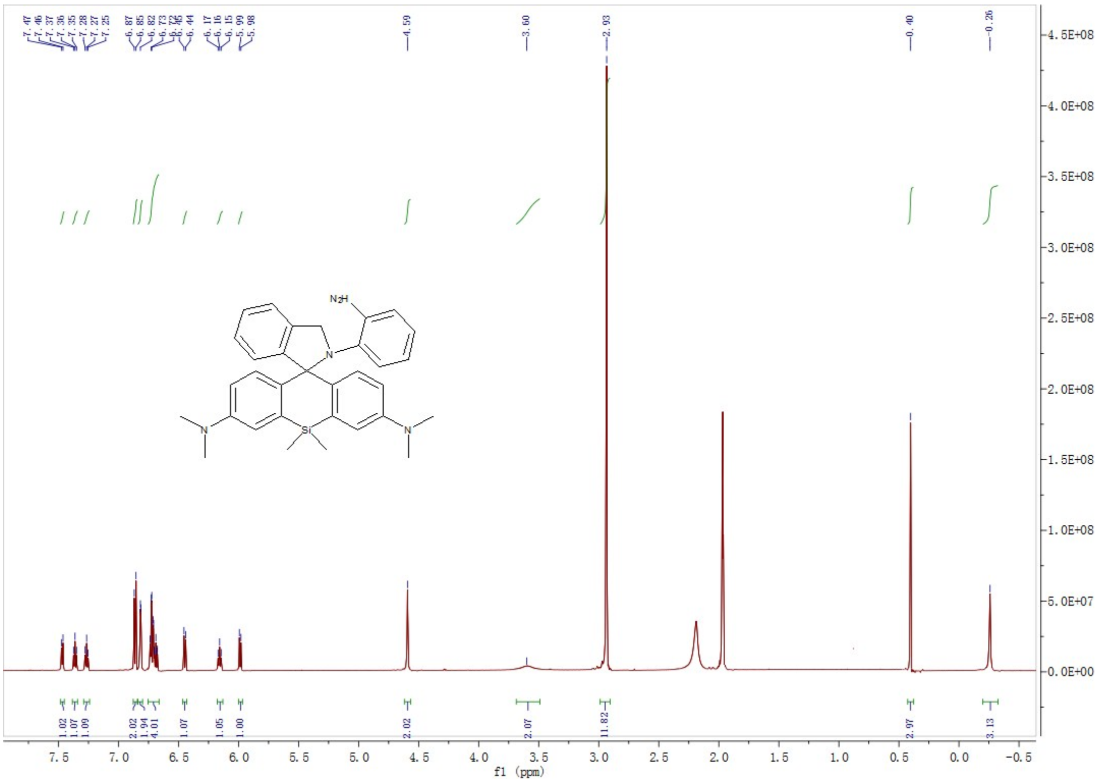

Figure S20 ^{1}H NMR chart of **deOxy-DALSiR** (CD_3CN, 600 MHz).

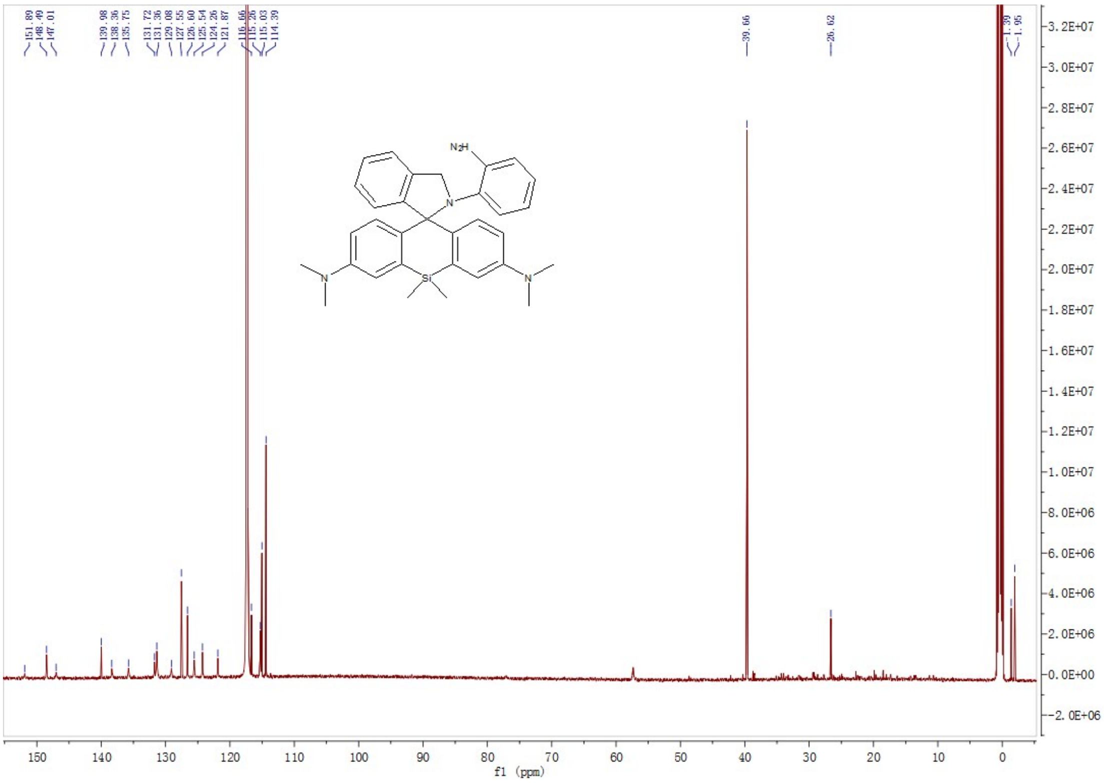

Figure S21 ^{13}C NMR chart of **deOxy-DALSiR** (CD_3CN, 600 MHz).

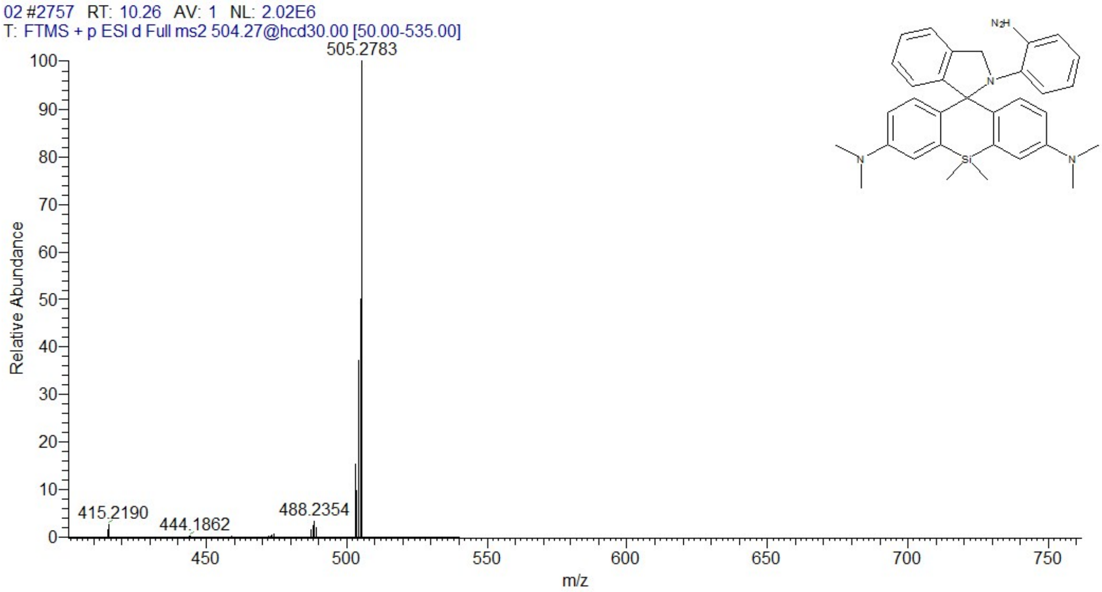

Figure S22 HRMS chart of **deOxy-DALSiR**.

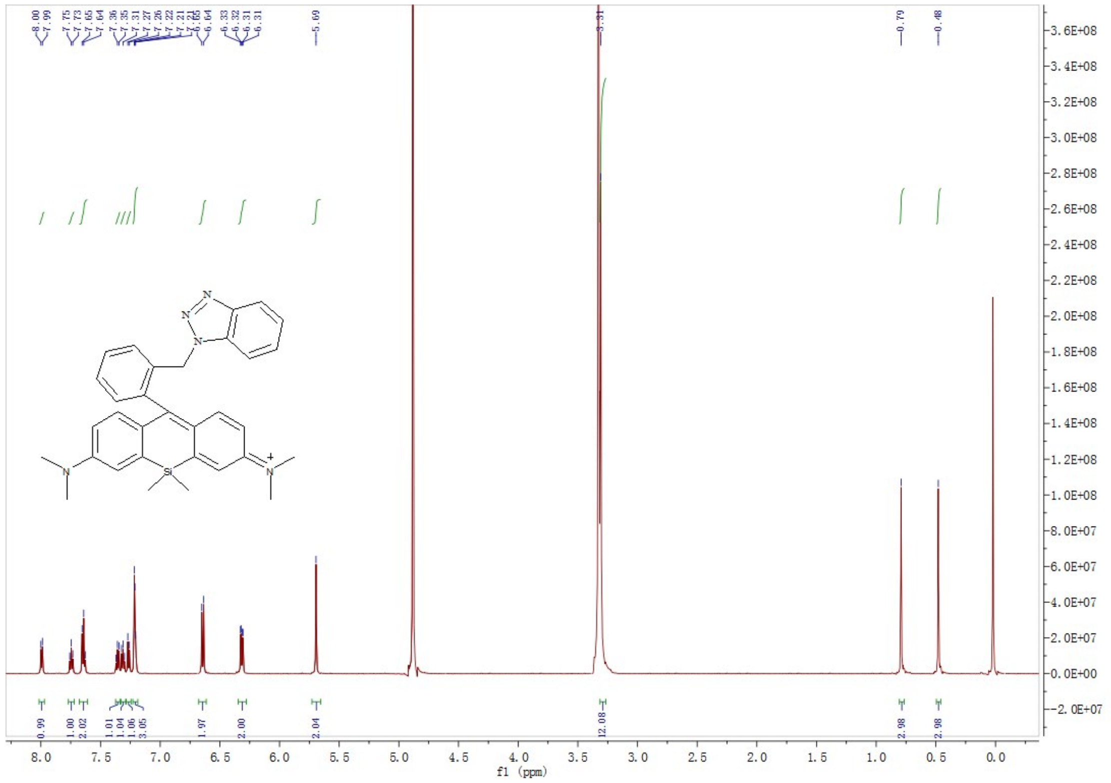

Figure S23 ^{1}H NMR chart of **deOxy-DALSiR-T** ($CDCl_3$, 600 MHz).

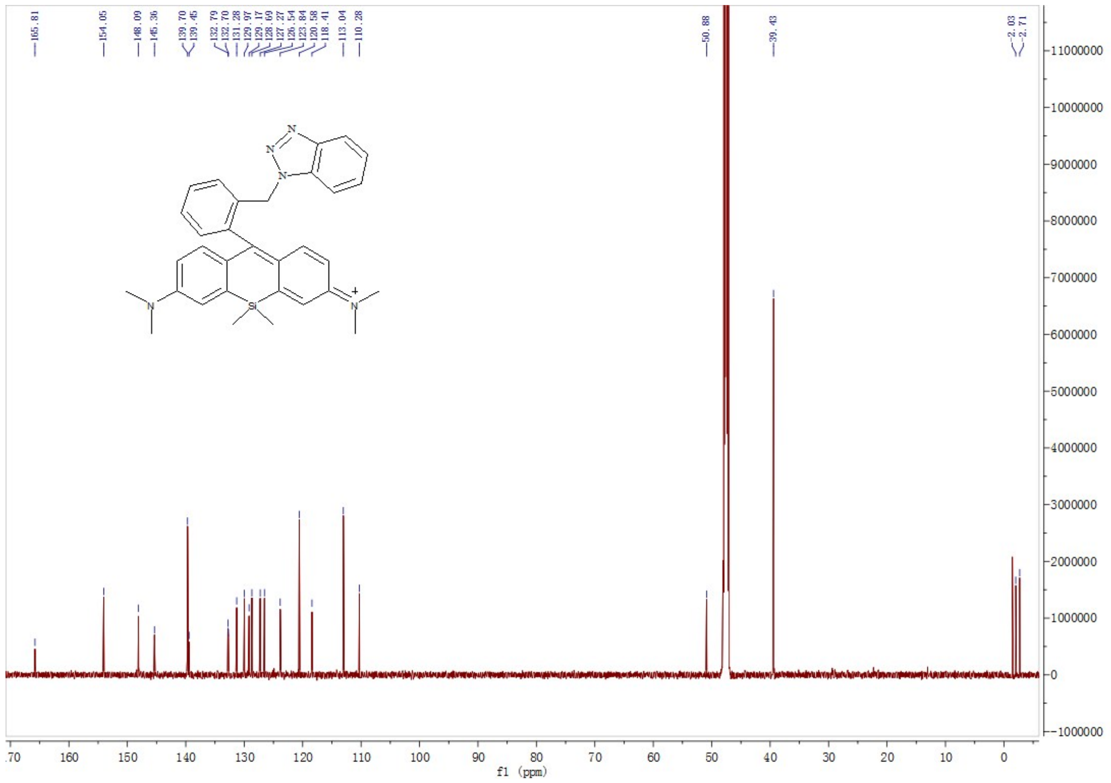

Figure S24 ^{13}C NMR chart of **deOxy-DALSiR-T** ($CDCl_3$, 600 MHz).

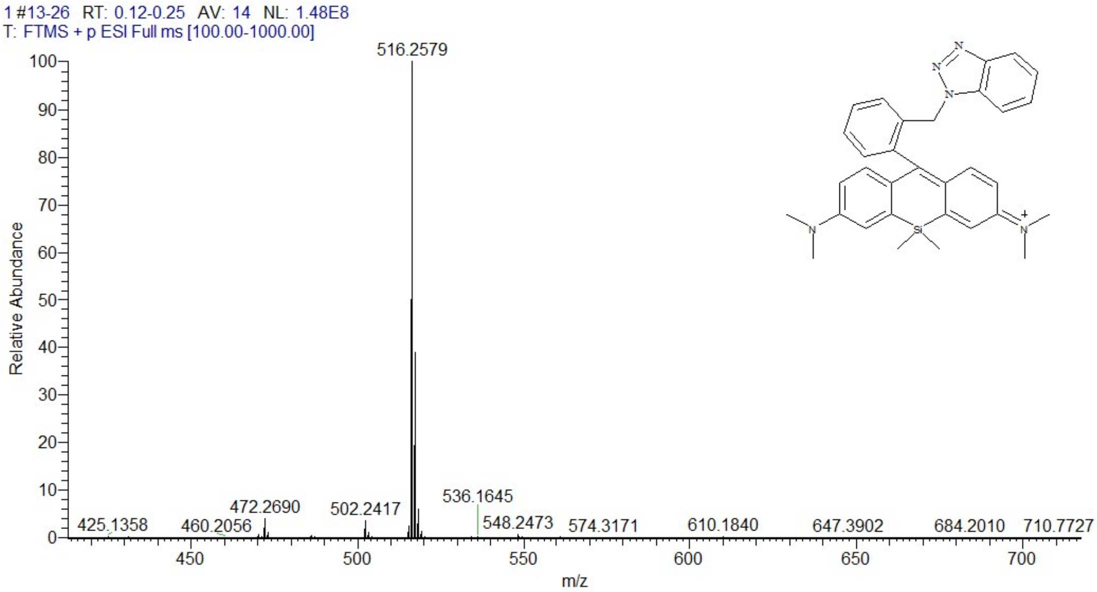

Figure S25 HRMS chart of **deOxy-DALSiR-T**.

9. References

1. H. Zheng, G.-Q. Shang, S.-Y. Yang, X. Gao, J.-G. Xu, *Org. Lett.*, 2008, **10**, 2357.
2. R. M Uppu, W. A. Pryor, *Anal. Biochem.*, 1996, **236**, 242.
